# Supplementary material for: The global polarisation of remote work
Source: PLoS One. 2022 Oct 20;17(10):e0274630. doi: 10.1371/journal.pone.0274630 (PMC9584402; doi:10.1371/journal.pone.0274630)
Supplement: S1 File — (PDF) [file pone.0274630.s001.pdf]

The global polarisation of remote work

— Supporting Information —

Fabian Braesemann[a,b,c], Fabian Stephany[a,b,c] Ole Teutloff[c,d], Otto Kässi[a,e], Mark Graham[a], Vili Lehdonvirta[a]

[a]Oxford Internet Institute, University of Oxford, Oxford, United Kingdom

[b]Humboldt Institute for Internet and Society, Berlin, Germany

[c]DWG Datenwissenschaftliche Gesellschaft Berlin, Berlin, Germany

[d]Copenhagen Center for Social Data Science, University of Copenhagen, Copenhagen, Denmark

[e]Etla Economic Research Helsinki, Finland

Correspondence: fabian.braesemann@oii.ox.ac.uk

September 16, 2022

Contents

S 1 Overview **2**

S 2 Conceptualising Remote Platform Work **2**

S 3 Empirical approaches to measure platform work **3**

S 4 Data collection and processing **8**

S 4.1 Collection of online platform data 9

S 4.2 Gecoding 11

S 4.3 Regional data sets 13

S 4.4 Occupational data 17

S 4.5 Occupation-level measures 22

S 5 Regression analysis of geographical polarisation **31**

S 5.1 Output feature 31

S 5.2 Explanatory features 33

S 5.3 Model specification 34

S 6 Additional analyses **38**

S 6.1 Robustness of regression results to data imputation 38

S 6.2 Analysis of model residuals 41

S 6.3 Sub-national granularity 42

S 6.4 Wage distribution across countries and occupations 43

S 6.5 Polarisation over time 44

References **49**

S 1 Overview

The supplementary materials contain all information relevant to reproduce the data collection, data preparation and the results presented in this study. In section S 2 we provide a conceptualisation of the term remote platform work. In section S 3, we list a number of relevant studies that have taken a similar empirical angle to the investigation of remote platform work and we describe their empirical setting and discuss the findings from these studies. While we can not publish the raw data itself due to privacy concerns, we explain the complete pipeline of our approach in a step-by-step description, including the discussion of problems we faced and ways we found to overcome them (section S 4). This description, together with the published code and aggregated data allow readers to reproduce all main results of the study and to adopt the methodology used here in future work. Where possible, we provide original data (aggregated on the regional or occupational level). In section S 5 we provide details on the choice of the regression models and their specifications. Section S 6 contains a number of additional analyses that we have undertaken to underline the robustness of the results presented in the main text and to provide complementary perspectives on some of the results.

Code and data are available on GitHub: `http://github.com/Braesemann/remotework`

S 2 Conceptualising Remote Platform Work

As a relatively new and highly diverse form of organising work, no clear agreement on terms and features related to remote platform work has emerged yet [1]. Commonly used names are: Crowdwork platforms [1, 2], online/digital labour markets [3–5], online platforms for contract labour [6, 7], freelance marketplaces [8, 9], online outsourcing platforms [10, 11] and online labour platforms [12, 13].

Three general characteristics about the concept of (remote) platform work emerge from reviewing the existing literature. First, labour is supplied by decentralized individuals from around the world and the product of their work is transmitted digitally. This explicitly excludes location-based gig work such as for example *Uber*, *Deliveroo* or *Book a Tiger*. Secondly, local wage differences and skill availability become accessible globally and thereby exploitable (coined "glocalization" by [14]). Much of today's platform work is in fact North-South transactions: employers from the global North hire relatively cheap labour from the Global South [6]. Thirdly, the decentralised workers are organized and matched to employers by third-party platform providers. This platform infrastructure represents the institutional framework which shapes the allocation of labour and capital [4]. Platform providers portray their role as intermediaries who enable employers and

workers to connect, denying any explicit employment relationship. However, platform providers implement and maintain a range of features and control mechanisms such as matching algorithms, reputation systems or payment infrastructure, which effectively equip operators with the capacities to shape how workers and employers interact. As a result, platform operators and platform design play a key role for market dynamics. This trend has been called 'platformization' [14]. In summary, remote platform work, as a new, entirely web-based form of organising work, is driven by *glocalization* (global exploitation of diverging local wage levels) and characterised by the *decentralization* of the workforce and the *platformization* of the worker-employer relationship. For the purpose of this study, we settle on the term *(remote) platform work* and *platform worker* as the most comprehensive but precise terms to describe the phenomenon under observation.[1]

There are a great variety of platforms that differ by the type of tasks, complexity of tasks, and the way work is allocated. This study uses data from a globally leading platform. To understand the position of this platform with the landscape of online labour, we build on the categorisation of remote work platforms developed by Schmidt (2017) [9]. According to this categorisation, platforms can be classified in cloud work (web-based) and gig work (location-based) depending on whether tasks can be completed via the internet or not. This study focuses on a web-based platform (*cloud work*). Platforms also vary depending on how tasks are distributed. *Crowd work* implies tasks being distributed to an undefined crowd of workers. Instead, Freelance or macrowork platforms refer to the distribution of tasks to specified individuals. Crowd work can be subdivided into microtasking crowd work and contest-based creative crowd work, depending on whether tasks are split up into small pieces equally paid for (microtasking), or whether workers compete against each other doing the same task and only the best results is used and paid for [9]. In contrast, on macrotask (freelance) platforms web-based tasks are given to selected individuals more akin to conventional labour markets. The platform data we investigate here contains macrotasks, not crowd- or click work. The platform is globally active with thousands of clients and several million platform workers from 180 countries. Tasks on the platform range from low skill (e.g data entry, administrative support) to high skill jobs (e.g. graphic design, software development) spanning all major types of jobs that can be performed via the internet [15]. In other words, the platform labour market investigated here serves as a case study for the general phenomenon of remote work, as it mimics conventional labour markets based on a fully digital infrastructure.

S 3 Empirical approaches to measure platform work

Here, we provide a detailed tabular overview of the key aspects of the studies that take an empirical approach to measure platform work, described in the corresponding section of the main text.

[1] To avoid repetition, we also use the term *online labour* interchangeably to *platform work* at several points in the text.

S Tab. 1 Review of empirical approaches to measure platform activity (part I).

Author & Title	Agrawal et al. (2015): "Digitization and the Contract Labor Market" [16]
Data & Method	Data from oDesk; descriptive statistics at the country level.
Key findings	The market for contract labor is growing. This online market is dominated by long distance north-south trade. Mean hourly wages differ significantly between countries ($4 in Philippines vs. $21 in China). However, online wages are higher than local minimum wages in all countries examined.
Limitations	Data from 2009 – 2013; unreliable national minimum wage data (Wikipedia estimates).
Author & Title	Hong and Pavlou (2017): "On Buyer Selection of Service Providers in Online Outsourcing Platforms for IT Services" [17]
Data & Method	Online labour platform data from corporate partner (name not disclosed); Fixed-Effects regression at the individual level (N = 117,105 workers).
Key findings	There is no level playing field in online labour. Employers have an aversion for service providers from countries with language, time zone and cultural differences; and a strong preference for workers from countries with high levels of IT development. Reputation could potentially overcome the negative effect of language and cultural differences (not time zone). Individual reputation could correct the bias towards workers from countries with high levels of IT development ('level the playing field').
Limitations	Limited to IT jobs one platform. No information on language information at the individual level.
Author & Title	Beerepoot and Lambregts (2015): "Competition in online job marketplaces: towards a global labour market for outsourcing services" [18]
Data & Method	Data from oDesk; Regression analysis at individual level (N = 925 workers).
Key findings	Global platforms act as a marketplace in which Western clients source work to contractors from developing countries, usually small jobs that are poorly remunerated. Wage convergence is noticeable. Workers from Western countries receive the highest absolute wages, but workers from developing countries receive the highest relative wages. However, experience and skills hardly translate into better remuneration. While service outsourcing via global online marketplaces provides new employment opportunities, the intense competition limits the financial gains for most contractors. The intense competition makes it difficult to build up experience on the platform.
Limitations	Only selection of countries (US, UK, India, and the Philippines). Very simplified operationalization of skill level (web development = high skill vs. administrative support = low skill).
Author & Title	Agrawal et al. (2016): "Does standardized information in online markets disproportionately benefit job applicants from less developed countries?" [19]
Data & Method	Data from oDesk; Regression analysis at the individual level (N = 356,480 workers).
Key findings	Employers from developed countries are less likely to hire workers from less developed countries even after controlling for a wide range of observables. Workers with standardized and verified work history information are more likely to be hired. Information on verified work history disproportionately benefits contractors from less developed countries. This premium also applies to additional outcomes including wage bids, obtaining an interview and being shortlisted. Informational limits to trade may be addressed through a variety of market design approaches; for instance, an online monitoring tool substitutes for verified work history information.
Limitations	—
Author & Title	Horton et al. (2017): "Digital Labour Markets and Global Talent Flows" [5]
Data & Method	Data from oDesk and Upwork; Regression analysis and gravity model (N = large but not specified).
Key findings	1) For work sent to India: Spatial distance, population levels, and telephone penetration do not matter in explaining labour flows. Strong role of ethnic diasporas (at least for India). 2) North-south nature of trade. Countries almost do not trade with themselves (except the US). 3) Less distance, common language and time zone boost contract placements 4) US employers are home-biased being more likely to hire expensive US workers 5) Limited substitution between the US and other countries: suggests that frictions to trade may still be quite persistent despite limited switching costs.
Limitations	Country-level only, the paper remains on a descriptive level.

S Tab. 2 Review of empirical approaches to measure platform activity (part II).

Author & Title	Ghani et al. (2014): "Diasporas and Outsourcing: Evidence from oDesk and India" [20]
Data & Method	Data from oDesk; Regression analysis at individual level (N = 35,000).
Key findings	Despite oDesk's efforts to minimize many trade frictions, diaspora connections still matter. Ethnic Indians are substantially more likely to choose a worker in India. There is a path-dependency in outsourcing with initial contracts being very important for long-term hiring habits. Taste-based preferences seem to play the largest role for the initial choice.
Limitations	Limited to a one-country case study (India). Would be promising to examine to what extent there are similar patterns with other ethnicities.
Author & Title	Rani and Furrer (2019): "On-Demand Digital Economy: Can Experience Ensure Work and Income Security for Microtask Workers?" [21]
Data & Method	Survey (N = 2350) on five global online labour platforms for microtasks (ATM, Figure Eight, Clickworker, Microworkers, Proflific); in-depth interviews with workers (N = 21); descriptive statistics.
Key findings	Despite high financial dependence on the work, returns to experience on the platform are meagre in terms of earnings, and highly experienced workers face the same risks as new entrants with regard to discrimination, high work intensity, lack of autonomy and control over work, and social protection. There is also a skills gap between the nature of tasks available on these microtask platforms and the workers' education levels. Finally, experience does not ensure that workers have the opportunities to undertake complex and challenging tasks, and the possibilities to develop their skills and improve career prospects are limited.
Limitations	Nothing on the statistical significance of the results presented in the paper. Limited to microtask platforms. Self-selection of survey respondents into the survey.
Author & Title	Anderson (2017): "Skill networks and measures of complex human capital" [8]
Data & Method	Data from Upwork; Network analysis and regression analysis (N = 26,046 worker profiles and 356,561 job listings).
Key findings	Workers with diverse skills earn higher wages than those with more specialized skills. There are two different types of workers benefiting from skill diversity: jacks-of-all-trades, whose skills can be applied independently on a wide range of jobs, and synergistic workers, whose skills are useful in combination and fill a hole in the labor market. On average, workers whose skills are synergistic earn more than jacks-of-all-trades.
Limitations	Data limited to a three-months period in 2013 – 2014. Many more interesting questions could be examined using the skill network approach.
Author & Title	Braesemann et al. (2020): "ICTs and the urban-rural divide: can online labour platforms bridge the gap?" [22]
Data & Method	Data source not disclosed ('globally leading online labour platform'); Regression analysis at the US county level (N = 3052 counties; 34,198 projects).
Key findings	Rural workers made disproportionate use of the online labour market. Rural counties also supplied, on average, higher-skilled online work than urban areas did. However, many of the most remote regions of the country did not participate in the online labour market at all. The findings highlight the potentials and limitations of such platforms for regional economic development.
Limitations	Limited to US data from 2013; relatively few data points.
Author & Title	Borchert et al. (2018): "Unemployment and online labor" [23]
Data & Method	Data from Microworkers.com; Regression analysis at the US commuting zone level (N = 657 commuting zones $\times$ 20 quarters = 13140 commuting zone-quarter observations).
Key findings	The findings highlight that many workers consider online labor markets as a substitute to offline work for generating income, especially in periods of low local labor demand. However, the evidence also suggests that, despite their potential to attract workers, online markets for microtasks are currently not viable as a long run alternative for most workers.
Limitations	Limited to one country.

S Tab. 3 Review of empirical approaches to measure platform activity (part III).

Author & Title	Lukac and Grow (2020): "Reputation systems and recruitment in online labor market: insights from an agend-based model" [24]
Data & Method	Data source not disclosed (one online labour platform); data used to confirm assumptions of agent-based model (N = 5000 projects); agent-based modelling (Simulations).
Key findings	The level of information asymmetry that characterizes different online labour platforms is one of the main determinants of how much inequality reputation systems will create.
Limitations	Very small sample size of empirical data to confirm assumption of agent-based model.
Author & Title	Lehdonvirta et al. (2019): "The Global Platform Economy: A New Offshoring Institution Enabling Emerging-Economy Microproviders" [25]
Data & Method	Data source not disclosed ('globally leading online labour platform'), 6 month data from 2013; N = 10,000 projects; Regression analysis at the project level.
Key findings	Individuals choose micro-providership when it gives a better return on their skills and labor than employment at a local (offshoring) firm. The platform acts as a signaling environment that allows microproviders to inform foreign clients of their quality, with platform-generated signals being the most informative signaling type. Platform signaling disproportionately benefits emerging-economy providers, allowing them to partly overcome the effects of negative country images and thus diminishing the importance of home country institutions.
Limitations	Limited to two occupations (Writing and Graphic Design) and employers from US and Canada.
Author & Title	Wood et al. (2019): "Good Gig, Bad Gig: Autonomy and Algorithmic Control in the Global Gig Economy" [26]
Data & Method	Data source not disclosed ('two globally leading online labour platforms'); Semi-structured interviews in six countries (N = 107) and a cross-regional survey (N = 679).
Key findings	Despite varying country contexts and types of work, they show that algorithmic control is central to the operation of online labour platforms. Algorithmic management techniques tend to offer workers high levels of flexibility, autonomy, task variety and complexity. However, these mechanisms of control can also result in low pay, social isolation, working unsocial and irregular hours, overwork, sleep deprivation and exhaustion.
Limitations	Small sample size for a survey in 6 countries; self-selection of workers into the interviews and the survey.
Author & Title	Lukac (2021): "Two worlds of online labour markets: Exploring segmentation using finite mixture models and a network of skill co-occurrence" [27]
Data & Method	Data from undisclosed OLM platform ('among the largest players in the field'). The web-scraper collected inputs on 12,123 projects with 188,622 bids, coming from 37,127 unique users from 172 countries (November 2019). Moreover, COLLEEM survey data is used represents an online panel survey on digital labour platforms, commissioned in 14 European countries (32,409 respondents in June 2017); finite mixtures of regression models, network analysis and clustering.
Key findings	Similarly to offline markets, online labour markets are composed of structurally delimited segments with different social processes governing the allocation of work. Mobility between segments in online platforms is limited. The segmentation explains large differences in the earnings potential of individual workers. Together, these results provide a new explanation for the persistence of diversified experiences in online labour markets and inform strategies for future research of online platforms as highly segmented labour markets.
Limitations	OLM dataset is relatively small and covers only one month.

S Tab. 4 Review of empirical approaches to measure platform activity (part IV).

Author & Title	Pallais (2014): "Inefficient Hiring in Entry-Level Labor Markets" [28]
Data & Method	Field experiment conducted on oDesk (Sample: 3,767 workers applying for data-entry jobs of which 50% were hired into the treatment group).
Key findings	Hiring inexperienced workers generates information about their abilities. If this information is public, workers obtain its benefits. If workers cannot compensate firms for hiring them, firms will hire too few inexperienced workers. This study determines the effects of hiring workers and revealing more information about their abilities through a field experiment in an online marketplace. The author hired 952 randomly-selected workers, giving them either detailed or coarse public evaluations. Both hiring workers and providing more detailed evaluations substantially improved workers' subsequent employment outcomes.
Limitations	—
Author & Title	Horton (2017): "The Effects of Algorithmic Labor Market Recommendations: Evidence from a Field Experiment" [29]
Data & Method	Experiment run by oDesk in 2011 (sample size for experiment: 6,209 job openings)
Key findings	Algorithmically recommending workers to employers for the purpose of recruiting can substantially increase hiring: in an experiment conducted in an online labor market, employers with technical job vacancies that received recruiting recommendations had a 20% higher fill rate compared to the control. There is no evidence that the treatment crowded out hiring of nonrecommended candidates. The experimentally induced recruits were highly positively selected and were statistically indistinguishable from the kinds of workers employers recruit "on their own." Recommendations were most effective for job openings that were likely to receive a smaller applicant pool.
Limitations	The experiment data is 10 years old. Given the high rate of innovation and expansion, platform design and dynamics might have changed significantly since 2011.
Author & Title	Stanton and Thomas (2015): "Landing the First Job: The Value of Intermediaries in Online Hiring" [30]
Data & Method	Data obtained from oDesk covering the period 1 August 2008 through 28 December 2009 (1126 intermediary agencies and about 150,000 workers); regression analysis.
Key findings	Online markets for remote labour services allow workers and firms to contract with each other directly. Despite this, intermediaries—called outsourcing agencies—have emerged in these markets. This article shows that agencies signal to employers that inexperienced workers are high quality. Workers affiliated with an agency have substantially higher job-finding probabilities and wages at the beginning of their careers compared to similar workers without an agency affiliation. This advantage declines after high-quality non-affiliated workers receive good public feedback scores. The results indicate that intermediaries have arisen endogenously to permit a more efficient allocation of workers to jobs.
Limitations	The data is more than 10 years old. Given the high rate of innovation and expansion, platform design and dynamics might have changed significantly since 2008/2009.
Author & Title	Kässi and Lehdonvirta (2019): "Do Digital Skill Certificates Help New Workers Enter the Market? Evidence from an Online Labour Platform" [31]
Data & Method	Data source not disclosed ('one of the largest online labour platforms'); N = 46,791 freelancers, 422,199 projects; regression analysis at freelancer and project level.
Key findings	The paper shows that obtaining skill certificates increases worker earnings. This effect is not driven by increased worker productivity but by decreased employer uncertainty. The increase in worker earnings is mostly realised through an increase in the value of the projects obtained (up to 10 %) rather than an increase in the number of projects obtained (up to 0.03 projects). On the whole, the results suggest that certificates play a role in helping new workers break into the labour market, but are more valuable to workers with at least some work experience. More stringent skill certification tests could improve the benefits to new workers.
Limitations	—

S 4 Data collection and processing

The data collection is an essential part of this study. One of the defining features of it is the large data set of online transaction records, which we have assembled and combined with other data sources. These data sets allow us to investigate the geography and skill polarisation of the remote labour market with more granularity than previous studies, which had to rely on small cross-sectional data sets gathered from web-scraping or other sources. In total, this study considers three types of data: (a) transaction records from a globally leading online platform, (b) regional covariates covering the demography, economy and infrastructure in OECD+BRIICS[2] and Global South countries, and (c) occupation statistics from the U. S. Bureau of Labour Statistics.

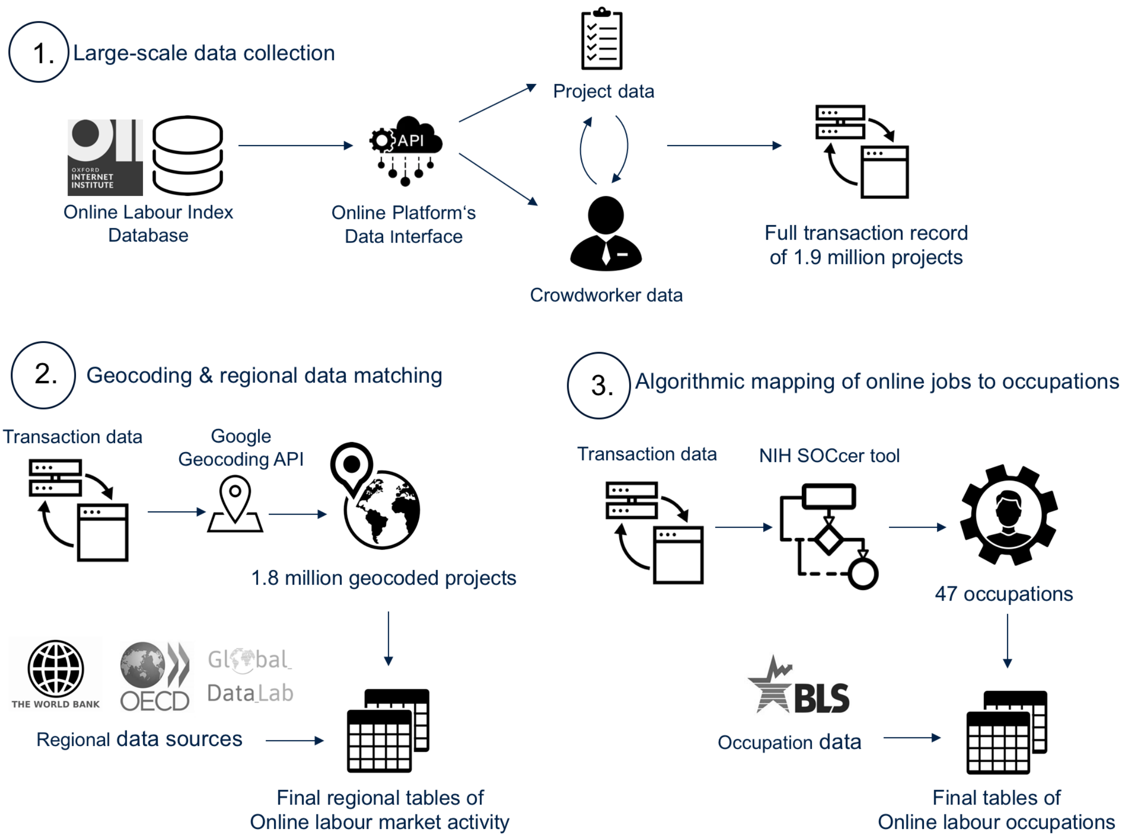

S Fig. 1 Illustration of the data collection and preparation. The data preparation consists of three main steps: (1.) data collection from the online platform, (2.) Geocoding of the platform data and matching with regional data sources, and (3.) mapping of platform job types to official occupation statistics.

Figure 1 visualises the main data source and the steps we have undertaken to prepare the data for the subsequent analysis. In summary, we have (1.) collected data from the online platform, (2.) geocoded the data and matched it to regional statistical databases, and (3.) we have mapped the online job categories to the Standardised Occupational Classification and merged the data with occupational statistics. All these steps are explained in more detail in the following sections.

[2]Brazil, Russia, India, Indonesia, China, and South Africa.

S 4.1 Collection of online platform data

Our analysis is based on transaction data from a globally leading platform for remote work.[3] As one of the largest global platforms [5], it features a great variety of jobs, ranging from relatively low-skilled data entry to more complex tasks, such as web design or software development. In contrast to microwork platforms (such as Amazon Mechanical Turk), the platform facilitates the coordination of larger projects that are generally of higher complexity than typical microwork tasks [15]. On the platform, workers apply for jobs posted by employers. Employers use the platform infrastructure to hire, monitor and pay workers. The job postings include a project title, a description of the task to be performed, a list of required skills as well as further formal requirements, such as formal contract duration or required language skills. Interested workers bid an hourly wage or fixed price on the open job postings. Before making a hiring decision, employers can interview applicants and review their public profile including working history and feedback from previous projects.

From the platform, we collected data in two ways. One dataset, which includes 330,000 transactions processed on the platform between March to August 2013 was provided to the Oxford Internet Institute directly from the platform in 2014. While this data set covers the entirety of all projects conducted on the platform in the observation period, it is limited by the relatively short time period. To gather more data from recent years, we additionally collected data using the database infrastructure of the Oxford Internet Institute's Online Labour Index [32].

The Online Labour Index (OLI) is the first economic indicator that provides an online gig economy equivalent of conventional labour market statistics. It measures the supply and demand of platform work across countries and occupations by tracking the number of projects and tasks across platforms in real time. To do so, it uses web-scraping to count the number of newly posted projects on a number of online labour platforms on a daily basis and it stores the individual project IDs.

We have used these project IDs and the Online Internet Institute's access to the online platform's database interface (API) to gather additional information, such as the project description and the employer's country-city location, about these projects (Fig. 1 panel 1). The project data, which we collected via the API, also contained information about the applicants to each project, including a unique platform worker ID. In total, the OLI database contained IDs of 4.8 million projects stored between 2016 and 2020. We could not retrieve information about all projects, as information about some of older projects were not available anymore, other projects did not contain publicly accessible information.

After having gathered information via the API about the projects of which we had IDs, we extracted the platform worker IDs from these projects. In a second step, we provided these IDs to

[3] The platform preferred not to be identified by name (for details see [25]).

the API in order to obtain the remaining information related to each transaction of these platform workers. This includes the hourly wage, the total price charged for the project, and the workers's country-city location. The individual platform worker profiles contain a project history (projects the platform workers had applied to in the past) ranging back to before the OLI data collection started in 2016. Thereby, we could obtain additional project IDs not yet covered in our dataset. In a third step, we collected the details about these projects, to fill the data gap between the transaction set from 2013 and the data collected from API requests covered by the OLI since 2016.

From the unique project IDs available in both the project and platform worker data sets, we could assemble a set of full transaction records. After removing incomplete observations and those that could not unambiguously be assigned to one platform worker, a data set of 1.6 million full transaction records remained. These were merged with the 330,000 projects from 2013, so that we end up with a complete transaction record for 1.9 million projects, covering the period from 2013 to 2020.

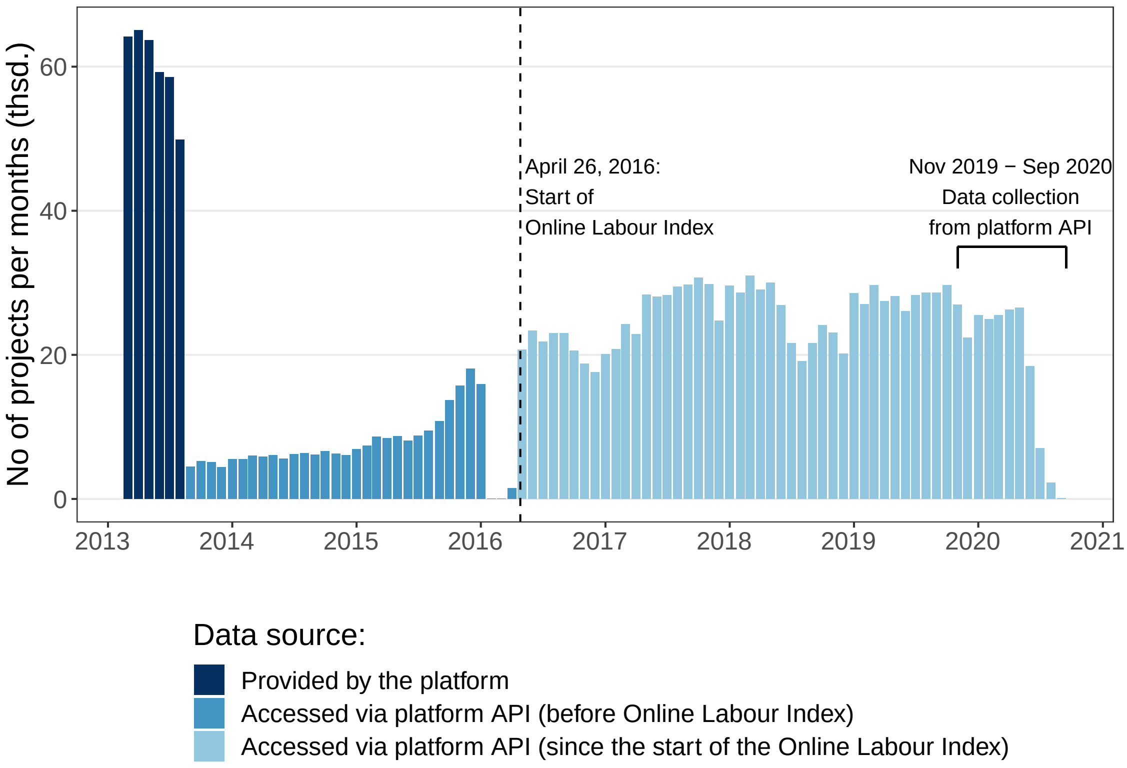

S Fig. 2 Number of projects per month collected from the online platform. The data comes from two sources: data of 2013 transactions directly provided by the platform (dark blue) and data collected via the platform's API (blue and light blue).

Figure 2 provides an overview of the size of the complete data set. It shows the number of projects per month collected from the platform. The data comes from two sources: the 2013

transactions, directly provided by the platform (dark blue), and data collected via the platform's API (blue and light blue). The 2013 data covers all transaction records conducted on the platform between March and August 2013, hence the larger volume during that period. From the data collected via the platform API, we distinguish between the projects that were directly captured in the OLI database since the start of the OLI data collection in April 2016 (light blue), and the projects from 2013 to 2015 that we collected in the third iteration of the API data collection (blue). The coverage of the data since May 2016 is roughly consistent up until summer 2020, when we finished the data collection, with only some seasonality. For the period from September 2013 to April 2016, we could collect less data, as we did not have access to all the daily project IDs, which were only made available through the OLI. Despite the differences in coverage between the years, we are confident that these do not affect the results of our analysis, as we do not compare the total number of projects between years (we use yearly fixed effects in the regression models to account for any differences in the data collection). Moreover, even in 2014 and 2015, where the data coverage is lowest, we still have data of 5,000 to 15,000 projects per month.

At this stage, we want to emphasise that we do not claim to cover the entirety of all projects conducted on the platform. We are aware that the OLI data collection captures only publicly advertised projects, hence it misses a share of projects, namely those that are privately assigned to platform workers [32]. Due to data accessibility limitations of the API, we could moreover not obtain full records of all the projects stored in the OLI database, even though the timing of the API requests did not seem to have influenced the number of projects we could collect (because of the roughly constant amount of data from May 2016 to May 2020). Nonetheless, we are confident that any accidental omission of data points was purely random. Hence, we assume that the data collection has not been systematically biased towards any of the variables of interest we are investigating here, that is country-city location or type of online job. This is moreover confirmed by the correlation of the overall geographical patterns reported in this study with those reported in other studies on the geography of the platform labour market (for example, [5]).

S 4.2 Gecoding

As outlined in Figure 1, we geocoded all transactions using the *Google Geocoding API*. To do so, we provide the Geocoding API wih a list of all unique country-city locations from both the employer and worker side of the platform transactions. We collected a total of 66,085 locations via the API. The geocoding is, theoretically, straightforward: free text is given to the Gecoding API (very much as if one was to perform a Google search query) and it returns longitude and latitude of the identified city centre together with the name of the country, city, and up to three sub-national levels: Admin level 1 corresponds to large regions, such as U. S. states, Admin level 2 corresponds to smaller regions such as U. S. counties, and Admin level 3 corresponds to municipalities.

In reality, however, there are a number of issues related to the algorithmic geocoding. Table 5 provides an overview of correct results of the gecoding (first five rows) and examples of errors (last five rows). Despite high data quality (platform workers have to verify their ID and location when registering to the online platform), not all locations are unambiguous. There are, for example, a number of cases where country and city location do not coincide. The location 'united states, geneva' (row 1962) is ambiguous (maybe platform workers or employers are active in both the United States and Switzerland). In the majority of such cases, the algorithm coded according to the city name, here Geneva in Switzerland as there is no Geneva in the United States. A second source of error is shown in the row 112: 'china, beijing' obviously means the capital of China, but there is also a restaurant called 'China Beijing' in Denver (`www.chinabeijingdenver.com/`), which the algorithm erroneously identified as the location searched for. A similar error occurred when different location have similar names. The location 'austria, wien' (row 632) obviously means the capital of Austria, but accidentally the algorithm identifies it as Vienna Township in Indiana. The other two types of potential errors are those cases, in which the algorithm fails to identify the administrative level correctly (row 146 'denmark, copenhagen'; row 173, 'russia saint-petersburg'): longitude and latitude are correct, but there are no identified sub-national regions or these are not properly displayed (in this case because of the Cyrillic letters).

S Tab. 5 Illustration of the results of the Geocoding algorithm and potential sources of error (66,085 unique locations in total).

Rank	Location	Count	Lon.	Lat.	Admin 1	Admin 2	Admin 3
1.	bangladesh, dhaka	69,162	90.41	23.81	Dhaka Division	Dhaka District	—
2.	united kingdom, london	59,856	-0.13	51.51	England	Greater London	—
3.	united states, new york	37,343	-74.01	40.71	New York	—	—
4.	india, chandigarh	35,004	76.78	30.73	Chandigarh	Chandigarh	—
5.	pakistan, lahore	33,076	74.35	31.52	Punjab	Lahore	—
112.	china, beijing	3,606	-104.91	39.66	Colorado	Denver County	—
146.	denmark, copenhagen	2,777	12.57	55.68	—	—	—
173.	russia, saint-petersburg	2,329	30.36	59.93	—	Ç-ü-µ-Ç-µ-Ä-±-É-Ä	—
632.	austria, wien	687	-85.77	38.65	Indiana	Indiana Scott County	Vienna Township
1962.	united states, geneva	210	6.14	46.24	Geneva	Geneva	—

To check the results of the geocoding and to correct errors, we have aggregated the results by the country provided from the platform users (first part of the 'Location' column in Table 5), together with the country and Admin 1 level information identified by the API. This resulted in a list of 3,269 locations, which was manually checked for any of these errors. Table 6 illustrates this for a number of cases. The first five rows show cases without an error.

The last five rows exemplify the three types of potential errors. In the case of 'copenhagen' and 'saint-petersburg' (see row 146 and 173 in Table 5), the country location has been correctly identified, but the sub-national level has not. To resolve this, the city-level element of the 'Location' column from Table 5 was entered as Admin 1 level. In the United Kingdom (see row 2 of table 5),

S Tab. 6 Table used to map with regional units from statistical databases (3,269 country-region combinations in total).

Country (platform)	Admin 1	Count	Country (API)	Correct	OECD region	GDL region
australia	Victoria	38,898	australia	True	Victoria	
argentina	Buoenos Aires	6,643	argentina	True		City of Buenos Aires
albania	Tirana County	1,289	albania	True		Tirana
algeria	Algiers Province	914	algeria	True		Nord Centre (Algier)
el salvador	San Salvador Department	968	el salvador	True		Central I
denmark	'copenhagen'	2,777	denmark	True	Copenhagen Region	
russia	'saint-petersburg'	2,329	russia	True	Federal City of Saint Petersburg	
united kingdom	Greater London	67,775	united kingdom	True	Greater London	
united states	Geneva	210	switzerland	False	Lake Geneva Region	
united kingdom	Tirana County	2	albania	False		Tirana

the API's Admin 1 level equals the countries of the United Kingdom, which is broader than the granularity used in OECD statistics. In these cases, the Admin 2 level has been inserted (see row 8 of Table 6). The last two examples of Table 6 show cases where country and city location do not coincide (see also the rows 112, 632, and 1962 in Table 5). In these cases, the comparison between the user provided country information and the one retrieved via API revealed the mismatch.

After having corrected all errors of the algorithmic geocoding, the country-region combinations are manually mapped to the regional units used in the regional statistical databases from the OECD and the Global Data Lab [33]. To do so, we used the Wikipedia article listing the administrative divisions of countries as the main source of reference.[4] The columns 'OECD region' and 'GDL region' in table 6 show examples.

Overall, the geocoding has not reduced the number of transaction records substantially, which could be used for the geographical analysis. Table 7 shows that only 0.3 % of the records could not be geocoded (at least to the country level). We end up with 1.868 million geocoded online labour projects. As not all countries in the data set are either being covered in the OECD regional database or in the GDL database, the total number of projects being considered in the regional analysis amounts to 1.835 million projects or 98 % of all projects.

S 4.3 Regional data sets

The geocoded online labour data is matched with national and sub-national statistics on demography, economy, and infrastructure. Three data sources are considered: World Bank for country-level statistics[5], OECD regional statistics for sub-national data in high and middle income countries from the Global North[6], and *Global Data Lab* [33] for sub-national level data for low and middle income countries from the Global South.[7]

[4] `http://en.wikipedia.org/wiki/List_of_administrative_divisions_by_country`
[5] `http://data.worldbank.org/`
[6] `http://stats.oecd.org/Index.aspx?DataSetCode=REGION_DEMOGR`
[7] `http://globaldatalab.org/areadata/`

S Tab. 7 Size of the final data sets used in the subsequent analysis.

Data Set	No or transaction records Absolute	Relative
**Total**	**1,873,462**	**100 %**
Geography		
Geocoded	1,868,466	99.7 %
Country-level	1,868,466	99.7 %
OECD regions	985,282	52.6 %
GDL regions	849,703	45.4 %
Occupations		
Occupation-level	1,869,309	99.8 %
Wage data	824,454	44.0 %

From all three sources, we have collected a variety of data sets that measure the following characteristics of each country or region: population size, education level, income per capita, internet connectivity, the IT specialisation of the local economy (measuring comparative advantages in IT-related industries or economic activities), English language capacity, and the price level.

Due to the different data sources, scope, and geography of each data set, there are differences in the exact coding of the measures. However, in all three data sources, we could identify measures that largely coincide. The individual data sets are listed in the following:

Population measures the size of the population per country or sub-national region. The measure is comparable across all three data sources. *Education* reflects the education level of the population in each area, measured by the share of children enrolled in secondary level education in the World Bank data (we hypothesised that, on a global level, differences between countries in secondary level education describe global education differentials better than those in tertiary level education), the share of people with a tertiary level educational degree in the OECD data, and by the average years of education in the GDL data. *Income per capita* is measured by GDP per capita in the World Bank and OECD data, and by Gross National Income in the GDL data. *Internet connectivity* represents the strength of the regional internet infrastructure and is measured by the share of fixed broadband subscriptions per population in the World Bank data, by the share of households with broadband access the OECD data, and by the share of households with internet access in the GDL data. The share of ICT exports of all service exports captures what we call the *IT specialisation of the economy* in the World Bank data. The concept is approximated by the gross value added in ICT in the OECD data. In the GDL data, there is, unfortunately not a corresponding measure. The IT specialisation of the economy is included to approximate the competitive strength of a country or region in IT-related economic activities and equally points towards an accumulation of specialised IT-skills. The *English language* variable indicates whether English is an official language in a country. It is, thus, only available on the national level. The data comes from

Wikipedia. The national *price level* reflects the purchasing power parity conversion factor to USD to capture differences in the purchasing power between countries. The data comes from the World Bank. The *Capital region* variable indicates whether a sub-national region holds the country capital. This last variable reflects differences between urban centres and other parts of each country and is particularly relevant in the Global South data set, where we lack variables capturing the IT specialisation of the local economy. A list of all the variables considered in the analysis and their coding are presented in Table 8.

In the regional statistical data sources, not all data points were available for all country-year or region-year combinations. In general, the data coverage was better in World Bank and OECD data than in the GDL data base. In order to not lose too many observations for the panel regression presented in Figure 2 of the main text, we decided to impute missing values.

This approach can be justified for two reasons. First, regional economic variables, such as the GDP per capita are relatively sticky. If a yearly observation between two other years is missing, it can be assumed the missing value will be close to the ones observed. Secondly, the aim of the regression models is not to derive a prediction of online labour project count or hourly wage within a region or country over time, but to establish a connection between online labour outcomes and regional economic and infrastructure variables on a global level. Thus, differences between countries or regions are considered more relevant than those within one country or region over time. Imputing some values of a region by others from the same region (or using the unconditional country average in those cases where no regional data is available) will maintain the differences between the regions, which are hypothesised to explain the differences in online labour market outcomes.

Because of these reasons, we imputed missing regional data points by others from within the same region (or country in case of World Bank data), used unconditional country averages where all regional data points were missing, and we imputed the 2019 and 2020 data points with 2018 values in all cases (as the regional data sets are published only with a time lag) in order to maintain as many regional data points as possible for the regression analysis. However, to validate that the data imputation did not affect the main results of the regression, we have also performed a regression with the original set of data points, as shown in Table 18 on page 40 below. Most coefficients show the same direction in these regression models, but due to less observations, some of the coefficients are (in contrast to the regression results shown in Figure 2 of the main text) not statistically significant. For more details on the data imputation and the robustness of the results with regards to the imputation of missing data points, see section S 6.1.

S Tab. 8 Coding of the regional variables considered in the analysis (WB: World Bank).

short var-name	long var-name	Source	Original variable
WB.POP.TOTL	Population, total	WB	Population, total
WB.SEC.NENR	School enrollment, secondary (% net)	WB	Ratio of children of official school age who are enrolled in school
WB.GDP.PCAP.CD	GDP per capita (current US $)	WB	GDP per capita (current US $)
WB.NET.BBND.P2	Share of fixed broadband subscriptions	WB	Fixed broadband subscriptions (per 100 people)
WB.GSR.CCIS.ZS	ICT service exports	WB	ICT service exports (% of service exports, BoP)
WB.NUS.PPP	PPP conversion factor	WB	PPP conversion factor, GDP (LCU per international $)
WK.ENG.LNG	English is de facto official language	WB	—
OLM.PRJ.CNT	Number of projects	WB	—
OLM.WG.MD	Median project wage	WB	—
OLM.WG.MN	Mean project wage	WB	—
OCD.PPL.CNT	Population count	OECD	Population, All ages
OCD.TED.PCT	Share of population with tertiary education	OECD	Share of population 25 to 64 year-olds by educational attainment: Total tertiary education (ISCED2011 levels 5 to 8)
OCD.GDP.PC	GDP per capita	OECD	Regional GDP: Millions USD, constant prices, constant PPP, base year 2015 / population count
OCD.BBD.PCT	Houshold share with broadband	OECD	Share of households with internet broadband access (in % of total households)
OCD.ICT.GVA	ICT Gross Value Added	OECD	GVA in information and communication (ISIC rev4): Millions USD, constant prices, constant PPP, base year 2015
OCD.CPT.YES	Region holds country capital	OECD	—
OLM.PRJ.CNT	Number of projects	OECD	—
OLM.WG.MD	Median project wage per hour	OECD	—
OLM.WG.MN	Mean project wage per hour	OECD	—
GDL.PPL.CNT	Population count	GDL	Total area population in millions
GDL.EDU.YRS	Mean years of education	GDL	Mean years education of adults aged 20+
GDL.INC.USD	Gross income per capita	GDL	Gross National Income per Capita (in 1000 US $ 2011 PPP)
GDL.IWI.IND	International Wealth Index	GDL	Mean International Wealth Index (IWI) score of region
GDL.INT.PCT	Household share with internet	GDL	% households with internet access
GDL.URB.PCT	Share of urban population	GDL	% population in urban areas
GDL.FRM.PCT	Share men in non-farm jobs	GDL	Percentage of employed men in upper nonfarm-jobs
GDL.CPT.YES	Region holds country capital	GDL	—
OLM.PRJ.CNT	Number of projects	GDL	—
OLM.WG.MD	Median project wage	GDL	—
OLM.WG.MN	Mean project wage	GDL	—

S 4.4 Occupational data

Besides the geographical analysis of online labour data, we also investigate the job types of the online projects. For this purpose, we match the online job categories with official occupational statistics used by the U. S. Bureau of Labour Statistics (BLS). The BLS provides detailed information about educational requirements, skills, and abilities of each occupation. This detailed data is available via the Occupational Information Network (O*NET).[8]

The job types used on the online platform do not necessarily correspond to official occupation titles. In many cases, they refer to job types that have developed in recent years in the digital economy, which do not have an equivalent in official occupational statistics. Others are related to business and professional services or to administrative support activities. To identify the occupation that most closely relates to each online job category, we map them to the 2010 Standard Occupational Classification (SOC). The mapping has been done in a semi-automatised way.

As shown in Figure 1, we used the *SOCcer* (Standardized Occupation Coding for Computer-assisted Epidemiological Research) tool provided by the U. S. National Institutes of Health [34]. The tool was developed to assist epidemiological researchers incorporate occupational risk into their studies. SOCcer takes the job title and job description as free text input and provides a list of SOC codes from the SOC 2010 classification system referring to occupations that match the description provided most closely. The tool is not intended to replace expert coders. Low scoring job descriptions indicate a weak fit with the suggested occupations and manual review is, in any case, needed to verify the coding. SOCcer uses an ensemble classifier combining multiple statistical classifiers to produce a single result. All the occupation codes suggested by the tool have been manually reviewed and the final mapping considered the detailed descriptions provided for each occupation by the BLS in the 2010 SOC Definitions.[9]

For the automatised coding with SOCcer, we used a sample of 345,000 online projects. We provided the online job category as job title, and the required skills and description of the online project as job description to the tool. SOCcer provides a list of ten potential occupations as result of the text analysis. This is because one job description can not be assigned unambiguously to one occupation, as the free-text information could describe several related types of occupations. Additionally, the results vary because of the different lengths of the job descriptions. Figure 3 illustrates this as it displays a histogram of the length of the job descriptions measured by the number of characters in each description. The length varies widely between online projects, and the histogram reveals a slightly left-skewed log-normal distribution with a $\log_{10}$ mean of 2.49 and a $\log_{10}$ standard deviation of 0.51.

To deal with the uncertainty induced by the algorithmic occupation classification, we calculated

[8] `http://www.onetonline.org/`

[9] `https://www.bls.gov/soc/soc_2010_definitions.pdf`

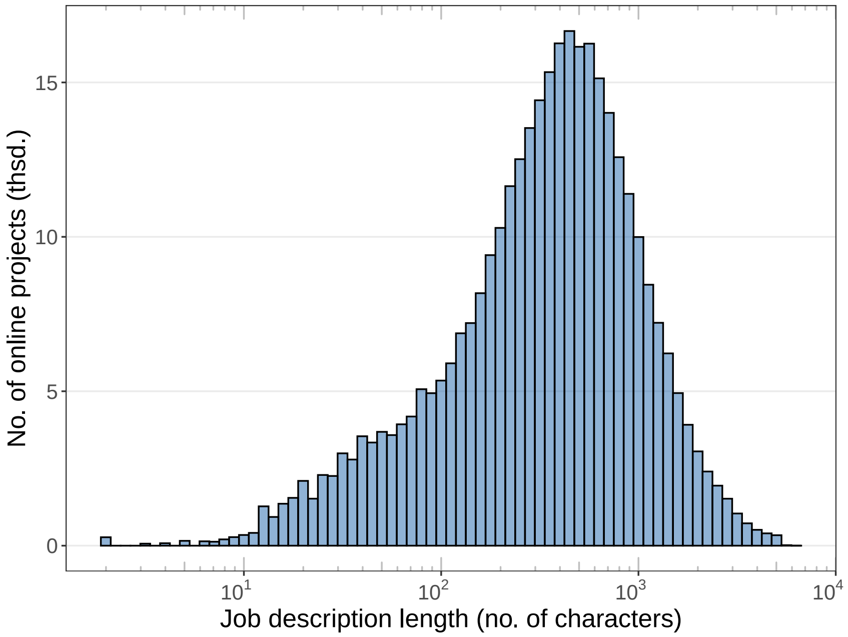

S Fig. 3 Distribution of job description length (no of characters) in sample provided to SOCcer tool. The description length varies widely between projects and follows a log-normal distribution.

the share of online projects assigned to each occupation by SOCcer per online job type and ranked the results. This was done for the first two suggested occupations from the tool (the first two suggested occupations were sufficient in all cases to find a reasonable match). Table 9 illustrates the coding of the occupations. The first five examples show cases where the algorithmic occupation classification obviously worked. For example, 88.7 % of all 'Data entry' online projects are identified as Data Entry Keyers (SOC code 43-9021). Similarly, 98.1 % of all 'Technical writing' jobs are identified as Technical Writers (SOC code 27-3042). These are obviously good suggestions and the job types were mapped to these occupations. The same holds for the job types 'Paralegal Services', 'Financial Planning' and 'Accounting', which were all assigned to a closely matching and reasonable occupation code.

Three other examples in the table show more difficult cases. For example, 82.9 % of the Web development jobs were, in the first instance, identified with the occupation Computer Programmers (SOC code 15-1131). This is not a bad fit. The description of the the occupation in the SOC Definition reads: '*Create, modify, and test the code, forms, and script that allow computer applications to run. Work from specifications drawn up by software developers or other individuals. May assist software developers by analyzing user needs and designing software solutions. May develop and write computer programs to store, locate, and retrieve specific documents, data, and information*'.

This definition is clearly related to the tasks being done by Web Developers, but their job focuses more on programming of websites, instead of stand-alone software development. To search

S Tab. 9 Illustration of the procedure applied to map online job categories to SOC occupations.

Online job type	SOCcer occupation	Rank	Share of jobs assigned (%)	SOC-code	SOC-occupation
Data entry	1	1	88.7	43-9021	Data Entry Keyers
Technical writing	1	1	98.1	27-3042	Technical Writers
Paralegal services	1	1	62.9	23-2011	Paralegals and Legal Assistants
Financial planning	1	1	96.5	13-2051	Financial Analysts
Accounting	1	1	74.6	13-2011	Accountants and Auditors
Web development	1	1	82.9	15-1131	Computer Programmers
Web development	2	1	37.3	11-3021	Computer & Information Systems Managers
Web development	2	2	34.5	15-1134	Web Developers
General translation	1	1	97.6	11-1011	Chief Executives
General translation	2	1	72.7	27-3091	Interpreters and Translators
Resumes & cover letters	1	1	58.5	51-4051	Metal-Refining Furnace Operators & Tenders
Resumes & cover letters	2	1	12.9	51-5112	Printing Press Operators
Resumes & cover letters	1	7	1.9	27-3043	Writers and Authors

for a better fit, we looked into the second most likely occupation being suggested by SOCcer. Here, the assignment is less clear. Around one third of the projects (37.3 %) is identified as Computer & Information Systems Managers (SOC code 11-3021) and a similar share (34.5 %) of projects is identified as Web Developers (SOC code 15-1134). The description of Computer & Information Systems Mangers ('*Plan, direct, or coordinate activities in such fields as electronic data processing, information systems, systems analysis, and computer programming*') describes managerial tasks, which are unlikely to describe the nature of the jobs being done by platform workers hired on 'Web development' online projects. Instead, both the title and the description of Web Developers ('*Design, create, and modify Web sites. Analyze user needs to implement Web site content, graphics, performance, and capacity. May integrate Web sites with other computer applications. May convert written, graphic, audio, and video components to compatible Web formats by using software designed to facilitate the creation of Web and multimedia content.*') fit the job very well. Hence, 'Web development' projects are mapped to SOC code 15-1134.

The two other examples in Table 9 illustrate cases with obvious mismatches. 'General translation' tasks are initially identified with Chief Executives (SOC code 11-1011). The tool seems to score heavily on certain keywords in the job title, here 'general'. As this is an obvious mismatch, we looked into the second column of the SOCcer suggested occupations, and found Interpreters and Translators (SOC code 27-3091) as the most frequent match, which is a good choice. A similar mismatch occurred with the next example. Tasks on 'Resumes & cover letters' ask platform workers to write CVs or update online resumes on websites such as *LinkedIn*. Therefore, Metal-Refining Furnace Operators & Tenders (SOC code 51-4051) or Printing Press Operators (SOC code 51-5112) are very unlikely to be a good fit. In this case, the best matching occupation

Writers and Authors (SOC code 27-3043) has not been matched often by SOCcer in the first or second column of the results; it is ranked only on position seven in the first column and on 15 in the second column of suggested occupations. Nonetheless, we assign the job to Writers and Authors, as the job descriptions of the online projects considered as examples largely fit to the official description of the occupation. Table 10 provides the SOC Definition of the assigned occupation and it lists two examples of online job descriptions. These give an overview of typical projects in these categories. We have used this approach for all online job types.

S Tab. 10 Examples of online project job descriptions in three categories and the best describing SOC definition.

Resumes & cover letters ⟶ SOC 27-3043 Writers and Authors	
SOC Definition	Originate and prepare written material, such as scripts, stories, advertisements, and other material.
Example 1	*' i'm at a loss of what to do with my linked in profile because of some shifts fortune 500 to small business and now want to head back into sales for a starup for fortune 500 so need my linkedin to show my all encompassing experience. i need to bring out my 20 years of corporate marketing in the introduction and then sprinkle in that i've been honing my skills as a business owner the past three years.'*
Example 2	*' i'm in the need of updating my linkedin profile and adding a professional background summary as well. the summary is to be no more then 250 words and end with a call to action. it also needs to tell a story of why i do what i do. i have a not to out of date resume and summary i have written myself as an example i can send. please advise f this is something you would be interested in.'*
Web development ⟶ SOC 15-1134 Web Developers	
SOC Definition	Design, create, and modify Web sites. Analyze user needs to implement Web site content, graphics, performance, and capacity. May integrate Web sites with other computer applications. May convert written, graphic, audio, and video components to compatible Web formats by using software designed to facilitate the creation of Web and multimedia content.
Example 1	*'we need 3 pages. 1. home page with slider and video 2. pricing page 3. blank page where we can add our own content.'*
Example 2	*'this job is to improve performance of a website and fix the bugs listed below. reduce the loading time of the website to less than 2 sec. fix errors in login contact us pages. load default collection file from webserver when the website is viewed every time in the free trail page. add seo to the website. include a close button to the home page video dialog box remove slide quotes tab buttons in mobile version. fix formatting issues in the free trail syntax tab. note the website uses 'themeforest' canvas template.'*
General translation ⟶ SOC 27-3091 Interpreters and Translators	
SOC Definition	Interpret oral or sign language, or translate written text from one language into another.
Example 1	*'3 x translation of 1 title, 5 bullet points, and a couple paragraphs. i have three more documents, like the last one, for translation. are you ready for more?'*
Example 2	*'hello, i have a document containing 5,597 words. some are repeated titles, describing different preventive safety measures for workers of a storage system. i provide a file with most technical terms that may appear in the document. the offer is 2cents word. i would need this to be delivered within 24 hours, and would love to find someone reliable i can work with long term, as i will be having more documents to be translated, with not so tight deadlines. thank you very much for bidding.'*

The cases shown in Table 9 illustrate that the occupation mapping will necessarily not be

unambiguous. Not only are there some occupations that are closely related to each other, making distinctions difficult, but there is always room for interpretation when analysing free-text information. Occupation mapping is different from geocoding: controlling for typing errors and language specific spelling, and assuming that users have not provided a fantasy location, user locations can be mapped to a clear set of country-city combinations (even though there are difficult cases, as we have seen in section S 4.2). In contrast, the vocabulary to describe job contents in the free-text information and online job titles varies more widely. Additionally, every occupation consists of tasks or requires skills that might be relevant in other occupations, too. Some online job types from the digital economy refer to occupations that did not yet exist when the occupation classification system was established, and these jobs can only be assigned to more generic occupations, such as 'Computer Occupations, All Other' (SOC code 15-1299). By accident, some employers might have assigned projects into categories that are a weak fit to the job type, and the algorithm comes with some flaws and biases, as shown in the case of 'General translation' projects. Because of these reasons, other coders will find a slightly different mapping more appropriate with some job types.

Despite these issues, we believe that there is value in the mapping online job categories to official occupations, as this approach allows us to investigate the differences between online job types using one established 'language'. Numerous studies have investigated the skill content of occupations (for example [35–37]) and they have used standardised occupation taxonomies. These are based on a well-researched and established methodology, and the mapping to SOC codes opens numerous possibilities to investigate online work and the changing division of remotely organised work in the digital economy.

Very much as the geocoding of our large-scale data set on the sub-national level described in the previous section allows us to investigate the global geography of the platform economy with an unprecedented granularity, the occupation mapping does the same with job types. We move away from the indistinguishable free-text job descriptions or ad hoc classifications based on the categories used by the online platform to a standardised and comparable taxonomy. The occupation mapping allows us to compare online jobs by educational attainment and standardised skill requirements. The mapping connects traditional occupation-based approaches to the study of labour markets with the platform economy. Based on the mapping methodology presented here, future studies could delve deeper into the evolution of skill requirements of platform work or the types of jobs being mediated online.

The mapping procedure was applied to all online job categories. The final mapping is displayed in Table 11 and 12. In total, 97 online job categories are mapped to 47 occupations. The SOC codes illustrate the different job types mediated via the platform. The majority is in Business and Financial Operations Occupations (ten job categories, SOC codes starting with 13-), Computer

and Mathematical Occupations (26 job categories, SOC codes starting with 15-), Architecture and Engineering Occupations (nine job categories, SOC codes stating with 17-), Legal Occupations (seven job categories, SOC codes starting with 23-), Arts, Design, and Media Occupations (29 job categories, SOC codes starting with 27-), and Office and Administrative Support Occupations (13 job categories, SOC codes starting with 43-). The mapping reveals a clustering on digital and ICT-heavy jobs as well as on professional and business services. What is common among all these jobs is that they, in some form, collect, process or mediate information. Thus, they can be conducted at a distance, even though some of these jobs (like Personal Assistants) would have been thought to require face-to-face interactions in the past. At this point, we want to emphasise again that other coders would have categorised some of the job types into different categories (for example Data Visualization to SOC 15-2099 rather than 43-9111). However, we are confident that the mapping reflects the main skill and task bundles associated with each job type. Therefore, we can use the occupation-level data made available via the mapping to understand differences in occupation-level outcomes on the online platform, such as wage per hours, which are relevant for platform workers.

S 4.5 Occupation-level measures

Based on the occupational mapping described in the previous section, we derived two measures capturing the skill or educational requirements of different occupations from BLS data and one capturing the relevance of experience in obtaining online projects.

Educational attainment score

The first measure is the educational attainment score (EAS), used in the occupation regression shown in Figure 3B of the main text. The score is a one-dimensional depiction of the educational differences between the occupations. In reality, one occupation does not require just one unique level of education, instead there will be people with different educational backgrounds. However, the distribution will differ between some jobs that do not require a particularly high level education and others that require more formal education. This difference is captured by the educational attainment score. The distribution of educational backgrounds for every occupation is provided by the Bureau of Labour Statistics. They are grouped into seven categories from 'No High School Diploma' to 'Doctoral Degree'.[10]

Figure 4 shows the distributions of eight occupations in each of the larger occupational groups. The average level of formal education varies substantially between the jobs. For example, the majority of Data Entry Keyers have a '*High School*' degree or '*Some College, but no degree*' as their highest level of education, while most Accountants have at least a '*Bachelor*' or '*Master*'

[10] `http://www.bls.gov/emp/tables/educational-attainment.htm`

S Tab. 11 Final mapping of online job categories to Standardised Occupational Classification (part I).

Online job type	SOC Occupation	SOC code 2010 (2018)
Management Consulting	Management Analysts	13-1111 (13-1111)
Project Management	Management Analysts	13-1111 (13-1111)
Email & Marketing Automation	Market Research Analysts and Marketing Specialists	13-1161 (13-1161)
Market & Customer Research	Market Research Analysts and Marketing Specialists	13-1161 (13-1161)
Marketing Strategy	Market Research Analysts and Marketing Specialists	13-1161 (13-1161)
Web Research	Market Research Analysts and Marketing Specialists	13-1161 (13-1161)
Brand Identity & Strategy	Market Research Analysts and Marketing Specialists	13-1161 (13-1161)
Accounting	Accountants and Auditors	13-2011 (13-2011)
Other - Accounting & Consulting	Accountants and Auditors	13-2011 (13-2011)
Financial Planning	Financial Analysts	13-2051 (13-2051)
Information Security	Information Security Analysts	15-1122 (15-1212)
Desktop Software Development	Computer Programmers	15-1131 (15-1251)
Game Development	Computer Programmers	15-1131 (15-1251)
Other - Software Development	Computer Programmers	15-1131 (15-1251)
A/B Testing	Software Developers, Applications	15-1132 (15-1252)
QA & Testing	Software Developers, Applications	15-1132 (15-1252)
Web & Mobile Design	Web Developers	15-1134 (15-1254)
Web Content	Web Developers	15-1134 (15-1254)
Web Development	Web Developers	15-1134 (15-1254)
Database Administration	Database Administrators	15-1141 (15-1242)
Network & System Administration	Network and Computer Systems Administrators	15-1142 (15-1244)
Technical Support	Computer User Support Specialists	15-1151 (15-1232)
Other - IT & Networking	Computer Network Support Specialists	15-1152 (15-1231)
Ecommerce Development	Computer Occupations, All Other	15-1199 (15-1299)
ERP / CRM Software	Computer Occupations, All Other	15-1199 (15-1299)
Lead Generation	Computer Occupations, All Other	15-1199 (15-1299)
Mobile Development	Computer Occupations, All Other	15-1199 (15-1299)
Other - Sales & Marketing	Computer Occupations, All Other	15-1199 (15-1299)
Other - Web & Mobile Development	Computer Occupations, All Other	15-1199 (15-1299)
Product Management	Computer Occupations, All Other	15-1199 (15-1299)
Scripts & Utilities	Computer Occupations, All Other	15-1199 (15-1299)
SEM - Search Engine Marketing	Computer Occupations, All Other	15-1199 (15-1299)
SEO - Search Engine Optimization	Computer Occupations, All Other	15-1199 (15-1299)
SMM - Social Media Marketing	Computer Occupations, All Other	15-1199 (15-1299)
Machine Learning	Data scientists and mathematical science occupations, all other	15-2099 (15-2051)
Quantitative Analysis	Data scientists and mathematical science occupations, all other	15-2099 (15-2051)
Chemical Engineering	Chemical Engineers	17-2041 (17-2041)
Contract Manufacturing	Industrial Engineers	17-2112 (17-2112)
Other - Engineering	Engineers, All Other	17-2199 (17-2199)
Architecture	Architectural and Civil Drafters	17-3011 (17-3011)
3D Modeling & CAD	Electrical and Electronics Drafters	17-3012 (17-3012)
Electrical Engineering	Electrical and Electronics Drafters	17-3012 (17-3012)
Mechanical Engineering	Mechanical Drafters	17-3013 (17-3013)
Other - Engineering & Architecture	Drafters, All Other	17-3019 (17-3019)
Civil & Structural Engineering	Civil Engineering Technicians	17-3022 (17-3022)
Physical Sciences	Physical Scientists, All Other	19-2099 (19-2099)
Contract Law	Paralegals and Legal Assistants	23-2011 (23-2011)
Corporate Law	Paralegals and Legal Assistants	23-2011 (23-2011)
Criminal Law	Paralegals and Legal Assistants	23-2011 (23-2011)

S Tab. 12 Final mapping of online job categories to Standardised Occupational Classification (part II).

Online job type	SOC Occupation	SOC code 2010 (2018)
Family Law	Paralegals and Legal Assistants	23-2011 (23-2011)
Intellectual Property Law	Paralegals and Legal Assistants	23-2011 (23-2011)
Paralegal Services	Paralegals and Legal Assistants	23-2011 (23-2011)
Other - Legal	Legal Support Workers, All Other	23-2099 (23-2099)
Art & Illustration	Fine Artists, Including Painters, Sculptors, and Illustrators	27-1013 (27-1013)
Illustration	Fine Artists, Including Painters, Sculptors, and Illustrators	27-1013 (27-1013)
Animation	Multimedia Artists and Animators	27-1014 (27-1014)
Physical Design	Commercial and Industrial Designers	27-1021 (27-1021)
Product Design	Commercial and Industrial Designers	27-1021 (27-1021)
Graphic Design	Graphic Designers	27-1024 (27-1024)
Graphics & Design	Graphic Designers	27-1024 (27-1024)
Motion Graphics	Graphic Designers	27-1024 (27-1024)
Presentations	Graphic Designers	27-1024 (27-1024)
Interior Design	Interior Designers	27-1025 (27-1025)
Voice Talent	Radio and Television Announcers	27-3011 (27-3011)
Public Relations	Public Relations Specialists	27-3031 (27-3031)
Editing & Proofreading	Editors	27-3041 (27-3041)
Academic Writing & Research	Technical Writers	27-3042 (27-3042)
Article & Blog Writing	Technical Writers	27-3042 (27-3042)
Grant Writing	Technical Writers	27-3042 (27-3042)
Other - Writing	Technical Writers	27-3042 (27-3042)
Technical Writing	Technical Writers	27-3042 (27-3042)
Content & Copywriting	Writers and Authors	27-3043 (27-3043)
Copywriting	Writers and Authors	27-3043 (27-3043)
Creative Writing	Writers and Authors	27-3043 (27-3043)
Resumes & Cover Letters	Writers and Authors	27-3043 (27-3043)
General Translation	Interpreters and Translators	27-3091 (27-3091)
Legal Translation	Interpreters and Translators	27-3091 (27-3091)
Medical Translation	Interpreters and Translators	27-3091 (27-3091)
Technical Translation	Interpreters and Translators	27-3091 (27-3091)
Photography	Audio and Video Equipment Technicians	27-4011 (27-4011)
Video Production	Audio and Video Equipment Technicians	27-4011 (27-4011)
Audio Production	Sound Engineering Technicians	27-4014 (27-4014)
Display Advertising	Advertising Sales Agents	41-3011 (41-3011)
Telemarketing & Telesales	Telemarketers	41-9041 (41-9041)
Customer Service	Customer Service Representatives	43-4051 (43-4051)
Other - Customer Service	Customer Service Representatives	43-4051 (43-4051)
Human Resources	Human Resources Assistants, Except Payroll and Timekeeping	43-4161 (43-4161)
Other - Admin Support	Secretaries and Administrative Assistants	43-6014 (43-6014)
Personal / Virtual Assistant	Secretaries and Administrative Assistants	43-6014 (43-6014)
Data Entry	Data Entry Keyers	43-9021 (43-9021)
Transcription	Word Processors and Typists	43-9022 (43-9022)
Logo Design & Branding	Desktop Publishers	43-9031 (43-9031)
Other - Design & Creative	Desktop Publishers	43-9031 (43-9031)
Data Extraction / ETL	Statistical Assistants	43-9111 (43-9111)
Data Mining & Management	Statistical Assistants	43-9111 (43-9111)
Data Visualization	Statistical Assistants	43-9111 (43-9111)
Other - Data Science & Analytics	Statistical Assistants	43-9111 (43-9111)

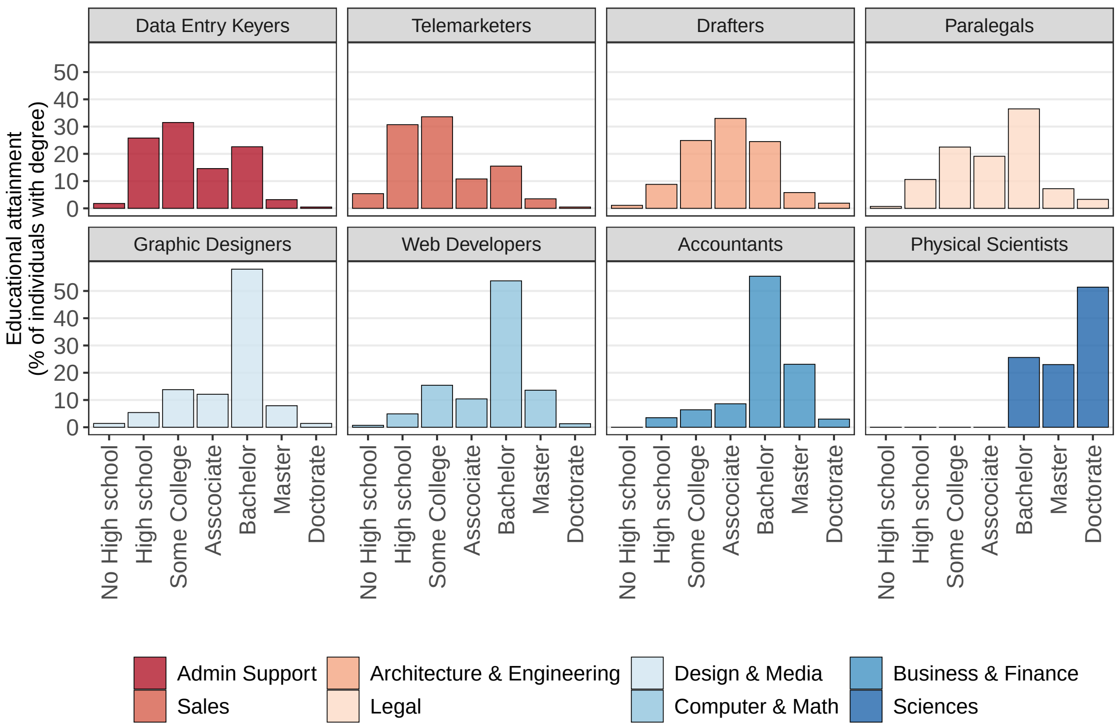

S Fig. 4 Distribution of educational attainment in selected occupations: The occupations in each of the eight groups vary in terms of their educational attainment distribution. From the share of individuals with with a given degree, we calculate the overall educational attainment score per occupation as a Likert scale.

degree. From the distribution of educational backgrounds, we calculate the estimated required educational level of each occupation as a single numerical score. We multiply the proportion of people at each educational level by Likert scale values from one (No High School degree) to seven (Doctoral degree). For example: 3 % of the Data Entry Keyers have no high school diploma (Likert value 1), 26 % have a diploma (2), 33 % have some college education (3), 14 % have an Associate's degree (4), 20 % have a Bachelor's degree (5), 4 % a Master's degree (6), and 1 % a Doctoral degree (7). Accordingly, the overall score of Data Entry Keyers is 48. The scores for Paralegals and Web Developers, for example, are 59 and 65, respectively. For comparability, we have finally transformed the variable to z-scores by substracting the mean and dividing by the standard deviation. The resulting score reduces the educational variety in different occupations, but it yields a common scale to compare the overall educational requirements between different occupations, which we use to investigate differences in occupation-level outcomes.

The scores of the 47 occupations vary substantially between the occupational groups (Figure 5). For example, the median score of the group 'Office and Administrative Support' is -1.54, while it is 0.37 for 'Computer and Mathematical'. The occupation 'Physical Scientists, All Other' (SOC code 19-2099) is at top of the scale with an educational attainment score of almost 3 points. Telemarketers (SOC code 41-9041) are at the bottom of the scale with -2 points.

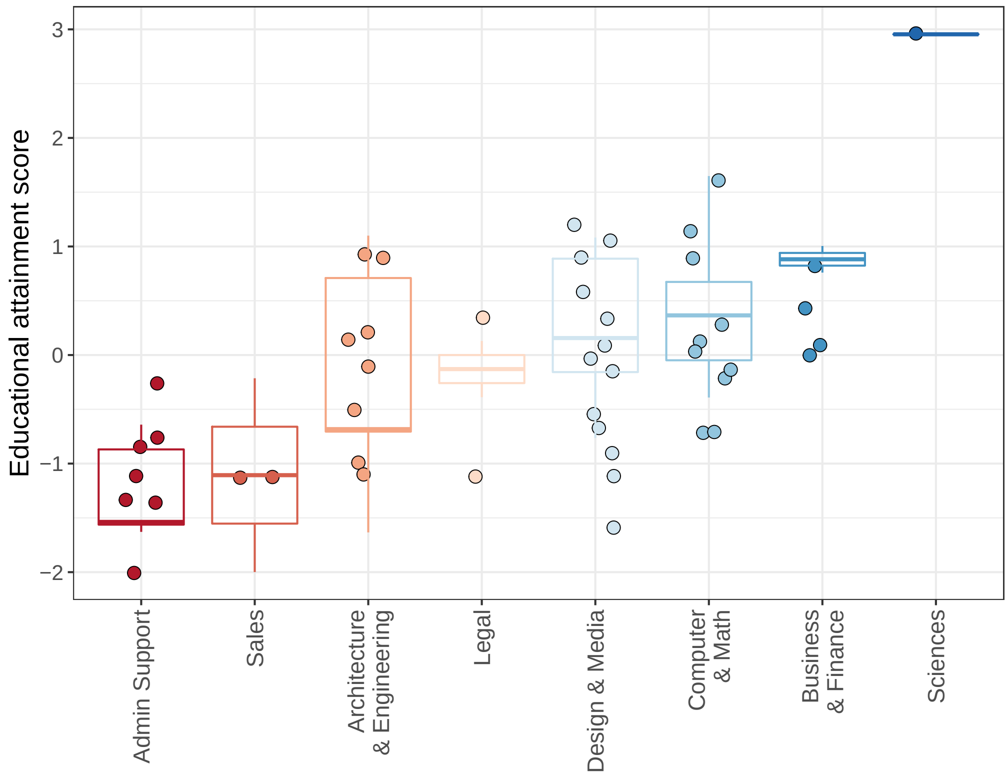

S Fig. 5 Educational attainment score in 47 occupations: The scores vary widely betwen occupations.

Skill cluster

Besides the educational attainment score, we present the distribution of skill requirements as a heatmap in Figure 3 of the main text. The data for the heatmap comes from the Occupational Information Network (O*NET) database 25.2.[11] We use the tables *Knowledge*, *Skills*, *Abilities*. These provide a mapping of O*NET-SOC codes (occupations) to Knowledge, Skill, and Ability ratings. The relevance of each type of knowledge, skill, or ability (for simplicity, we call all three types together 'skills') is displayed on a scale between one and five. The data contains a total of 119 skills. To reduce the noise in the data for display in the heatmap, we have filtered only those skills that have an average importance of at least 2.5 points among the 47 occupations. This reduces the number of skills to 55.

To group similar skills and occupations, we use the hierarchical cluster algorithm that comes with the '`pheatmap`' R-package.[12] The algorithm uses the Euclidean distance measure and complete linkage as clustering method. After having tried different specifications, we did cut of the dendrogram to form six occupation clusters and nine skill clusters. Different choices would have been possible at this stage, but they would not have majorly altered the identified differences

[11] `http://www.onetcenter.org/database.html#all-files`
[12] `http://cran.r-project.org/web/packages/pheatmap/pheatmap.pdf`

between the groups of occupations displayed in the heatmap.

Experience gradient

In the occupation analysis presented in Figure 3 of the main text, we relate occupation-level variables to the importance of experience in obtaining online projects. This is what we call the '*experience gradient*'. The idea is that experience and reputation are known to drive outcomes in the platform economy, as they signal trustworthiness of sellers [38–41]. This is important for platform workers, who are not just aiming to obtain high wages, but who also want to obtain projects in the first place. The importance of reputation or experience most likely varies between the occupations, for example because the highly specialised skills required in certain types of jobs already function as a trust cue, or because relatively low labour supply in certain occupations does not give employers much choice in hiring platform workers based on their experience.

As a way to operationalise the differences in the relevance of previous experience or feedback between occupations, we calculate the experience gradient as the slope parameter estimate $\hat{\beta}$ from a regression model of the yearly project count per platform worker on the total experience of the same platform worker. Table 13 and Figure 6 illustrate this. From the 1.87 million projects conducted by 393 thousand platform workers in the data set, we construct a panel on the worker-year-occupation level, counting the number of projects a platform worker has worked on per occupation in year t and the total experience measured by the number of projects conducted in previous years from the registration of the platform worker until the year $t-1$ in all occupations. For example, platform worker A in Table 13 worked on four projects in 2017. Together with the one project he completed before 2017, he has a total experience of five projects in 2018.

S Tab. 13 Illustration of the data used to estimate the experience gradient.

Platform worker	Year$_t$	SOC code	Project count	Platform worker experience$_{(t-r\,\ldots\,t-1)}$
A	2018	13-1111	1	5
A	2018	15-1151	7	5
A	2017	15-1151	3	1
A	2017	27-3091	1	1
B	2018	43-9022	1	1
B	2017	43-9022	1	0

From this panel data, we extract a random sample of 1,000 observations per occupation and perform a simple linear regression of the project count (y) on the platform worker experience (x).[13] Figure 6 shows this for four occupations. These simple models are an obvious oversimplification of the real relationship between the number of projects a platform worker has performed in any given

[13] We use samples of 1,000 observations in order to make sure that the different sample sizes between smaller and larger occupational groups itself does not influence the regression results. The sampling is with replacement. This allows to draw samples with 1,000 observations also from those occupations that have a smaller sample size.

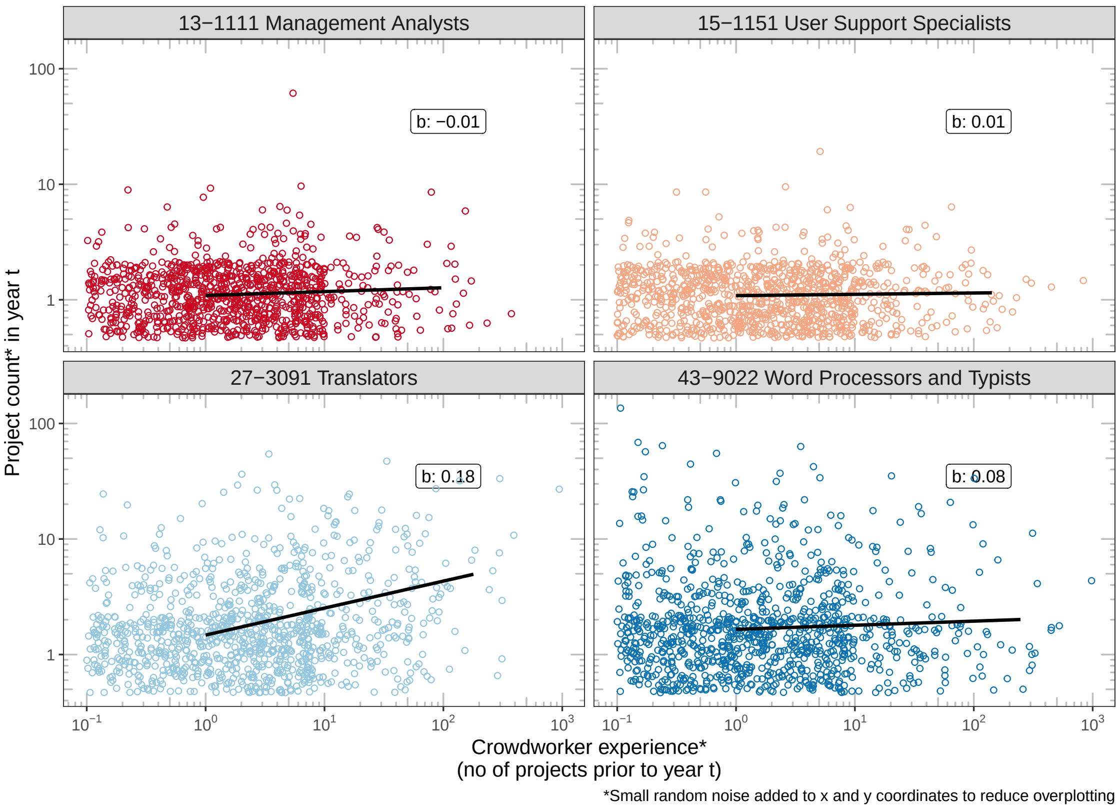

S Fig. 6 Illustration of the regression yielding estimates of the experience gradient. The values vary substantially between occupations.

year and worker-level characteristics. In the model, we do not control for unobserved heterogeneity among the platform workers (via fixed effects), nor do we include any other covariates. Nonetheless, the model is parsimonious and the $\hat{\beta}$ values between the 47 occupations vary substantially. For example, the $\hat{\beta}$ of User Support Specialists (SOC code 15-1151) in Figure 6 is 0.01, while it is 0.18 for Translators (SOC code 27-3091).

These differences point towards differences between the occupations in the relevance of prior experience in explaining the project count per platform worker and year. In other words, prior experience is not valuated the same way in the different occupations. In some types of occupations, prior experience is a more relevant trust cue than in others. These differences can be explained with occupation-level variables, such as the size of occupational sub markets[14] (measured by the total number of projects per occupation in the online labour market) and average wages, as we show in Figure 3 of the main text.

In order to make sure that the results of regression model (2) in Figure 3B of the main text are not biased by the random sample of 1,000 observations per occupation to derive an estimate of the experience gradient, we have repeated this exercise 1,000 times. This repeated sampling yields a

[14]But only for certain types of occupations, those that we label 'Non-Tech' in Figure 3 of the main text.

sample distribution of the experience gradient for each occupation. These are shown as boxplots in Figure 7. The sampling used to derive the experience gradient, indeed, influences the result, as can be seen by the variation of each boxplot. Nonetheless, the overall differences in the median values becomes obvious. While the occupations at the bottom of the distribution show estimated experience gradients of around zero, the ones in the middle of the distribution show median values of around $0.05 - 0.1$, and the occupation at the top (SOC code 27-3091) shows average values of 0.2.

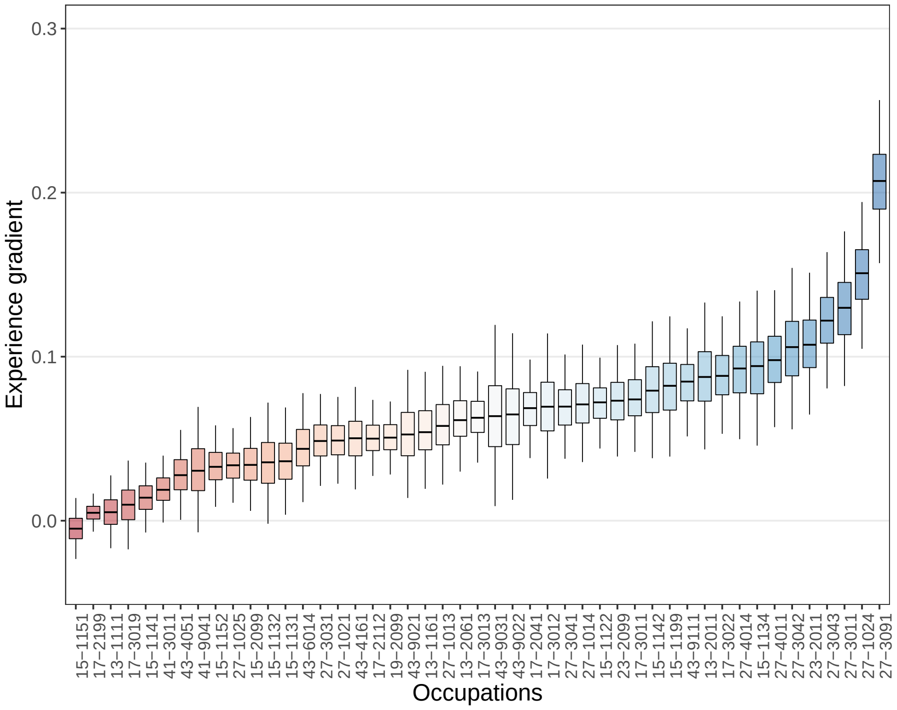

S Fig. 7 Distribution experience gradient estimates from 1,000 repeated samples per occupation.

The 1,000 estimates of experience gradients allow us to relate them to occupation-level variables (see Fig. 3B of the main text) in two ways. We can either relate the median values of the experience gradients (shown in Fig. 7) to occupation-level variables in one regression model, or we can conduct 1,000 regression models relating each estimate of the experience gradient with occupation-level variables and present the median values of the coefficients, standard deviations, and goodness-of-fit values from all regressions. Table 14 compares both approaches. It reveals that the results are almost identical, the directions and magnitude of the coefficients are very similar.

The distribution of the main values from the 1,000 regressions of model (2b) in Table 14 is shown in Figure 8 (mean values are highlighted as vertical lines). The varying goodness-of-fit is shown in the fourth row. The majority of models is able to explain between 20 % and 40 % of the variation, but the best performing models have R^2 values of more than 0.5 (see also the 5 % and 95 % quantiles of the R^2 estimates in Table 14).

S Tab. 14 Regression models relating the experience gradient to occupation-level variables. Model (2a) presents results from *one* model; the y-variable equals the median values of 1,000 estimated experience gradients (see Figure 7). Model (2b) presents the median values of 1,000 regression models, each relating one estimated experience gradient to occupation-level variables together with the 5 % and 95 % quantile. Both models do not differ in the direction or magnitude of the coefficients, but the overall goodness-of-fit varies.

Dependent variable:	Experience gradient[a,b]			
Model	(2a)	(2b)		
	Regression of Exp-grad. medians	Median	1,000 regressions: 5 % quant.	95 % quant.
Avg. no. of applicants	-0.01	-0.01	-0.03	0.001
(log-transf.)	(0.02)	(0.02)	(0.02)	(0.02)
Market size	**0.02*****	**0.02*****	0.01	0.02
(avg. project count, log.)	(0.004)	(0.004)	(0.003)	(0.005)
Educational attainment	-0.01	-0.04	-0.08	0.004
score (EAS)	(0.01)	(0.05)	(0.05)	(0.06)
Average wage	**0.04*****	**0.04*****	0.03	0.05
(log-transf.)	(0.01)	(0.02)	(0.01)	(0.02)
Constant	-0.2^{***}	-0.2^{**}	-0.3	-0.1
	(0.08)	(0.08)	(0.07)	(0.09)
Observations	46	46		
R^2	0.37	0.35	0.26	0.44
Adjusted R^2	0.30	0.29	0.18	0.0.39

Note: *p<0.1; **p<0.05; ***p<0.01

[a]Log-transformed.

[b]Equals $\hat{\beta}$ from an occupation-wise regression of projects per worker in a given year on no. of projects in previous years per worker.

Independently of the concrete specification of the regression model (2) relating the estimates of the experience gradient with occupation-level variables, the coefficients point into the same direction. The repeated sampling procedure presented here underlines the stability of the results: in certain occupations (those that tend to be larger markets with more projects overall, and those that tend to payer higher average wages) previous experience is more relevant for platform workers to obtain projects than in others.

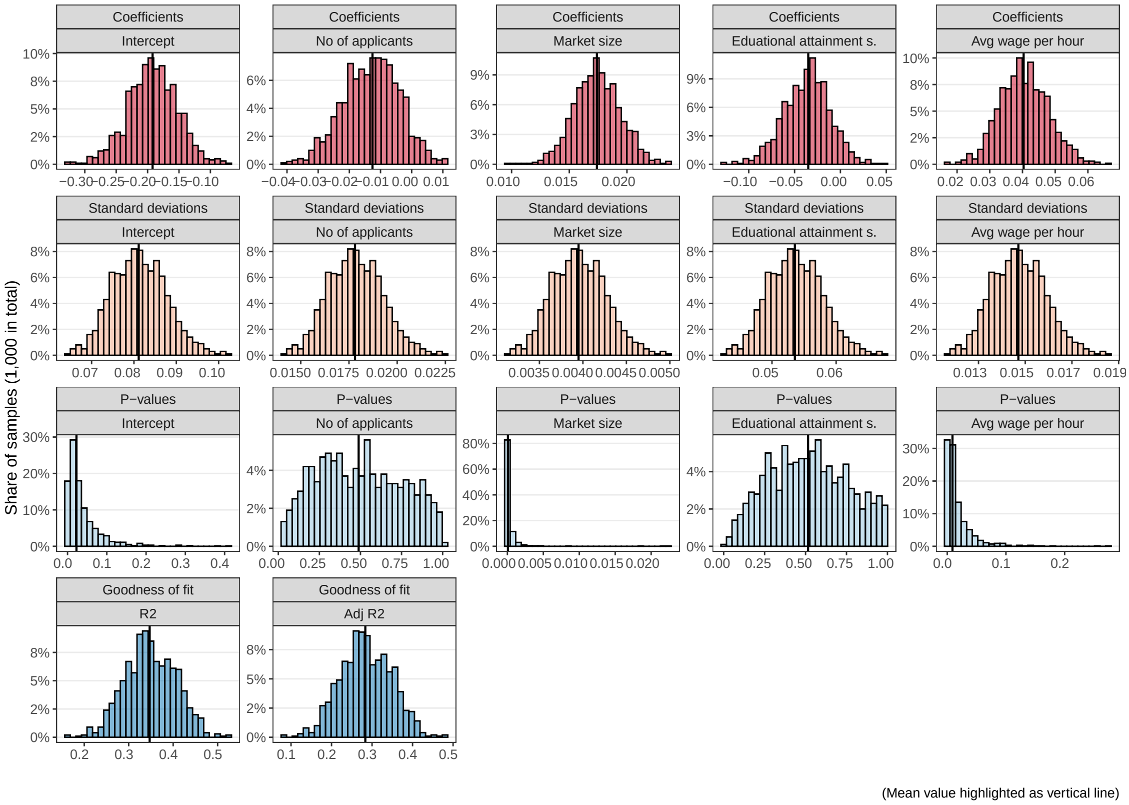

S Fig. 8 Distribution of coefficients, standard deviations, p-values, and goodness-of-fit measures from regression model (2) in Fig. 3B of the main text, based on 1,000 repeated samples.

S 5 Regression analysis of geographical polarisation

The regression analysis of the geographical distribution of online labour projects and wages relies on six regression models, where we regress a broad set of regional characteristics on regional and country-level wages and project count. Before conducting the regression analyses, a number of preparations have to be done, as we outline in the following sections.

S 5.1 Output feature

The target features of our regression model, a country' or region's project count / mean wage have unique properties that require a transformation before inferential analysis.

Figure 9, for example, shows the distribution of the online labour project count across countries. The distribution of projects is extremely right-skewed and has many zero values: the mean of the distribution is significantly higher than its median and 18 percent of all regional project counts are zero.

The underlying project and wage samples in the other two data sets (country-level and Global South regions) show a similar distribution pattern, as summarised in Table 15, with wages being less extremely skewed than project counts.

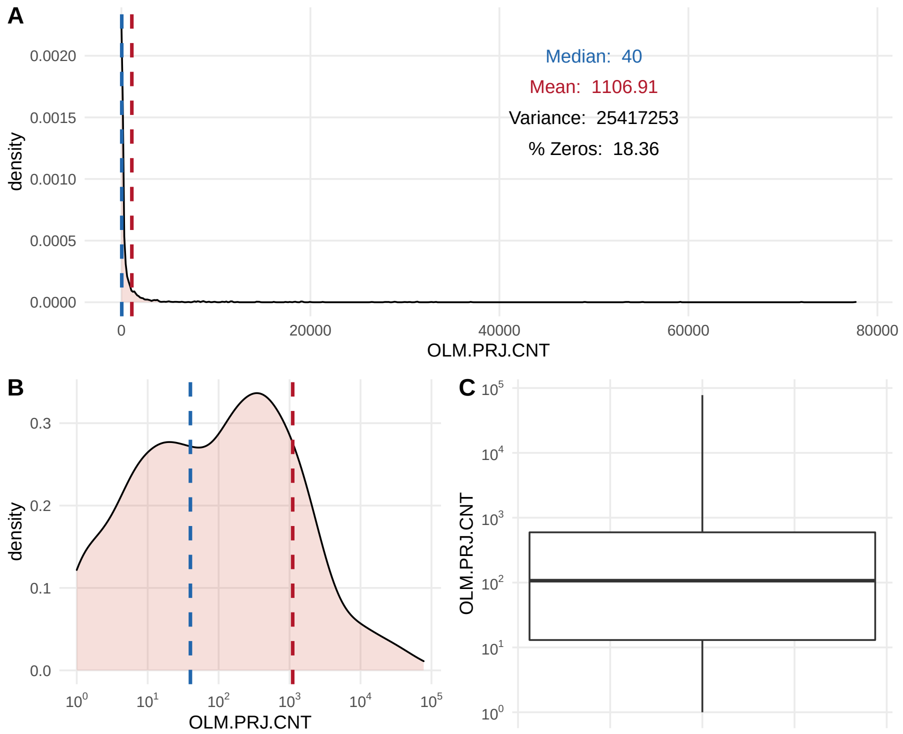

S Fig. 9 Distribution of projects across countries. **(A)** Density of the project count on a non-transformed scale (blue line: median, red line: mean): a huge variation becomes apparent. **(B)** Density of the project count on a $\log_{10}$ transformed scale. **(C)** Distribution of project count as boxplot on a $\log_{10}$ scale.

S Tab. 15 Distributions of project counts and mean wages are right-skewed and include many zeroes.

Data Set	Distribution features		
	Median	Mean	Zeros
Project Count			
Country	40	1.107	18 %
OECD+	27	186	17 %
GDL	0	127	58 %
Avg. Wage			
Country	18	23	23 %
OECD+	21	24	25 %
GDL	13	39	64 %

To adjust for the distributional features of our data, various transformations of the output features, project count and median wage are possible. A logarithmic transformation adjusts for the right-skewed pattern of the distribution. However, this approach would require to drop all zero value observations, which make a substantial part of the sample. To maintain the full sample size (including zero values) and still adjust for the uneven distribution of the data, we consider an inverse hyperbolic sine (ihs) transformation: $y = log(x + \sqrt{x^2 + 1})$ [42]. Alternatively, we could perform our regression analysis under the assumption that our target feature has a negative binomial distribution and succeed without any previous transformation. For the subsequent model

choice, we consider all three options and compare their out-of-sample accuracy with a full set of features, as described in the subsequent section.

In addition to the question of output feature transformation, we would like to assure a consistent representation of regional average wages. For regions with small project sample sizes, the variance in wages is substantial, as shown in Figure 10. The mean wage has strongest dispersion for regions with a project count below 25. We therefore limit our wage regression sample to regions with a sample size of at least 25 projects in a given year. This reduces the potential effect of outliers on influencing the wage regression models.

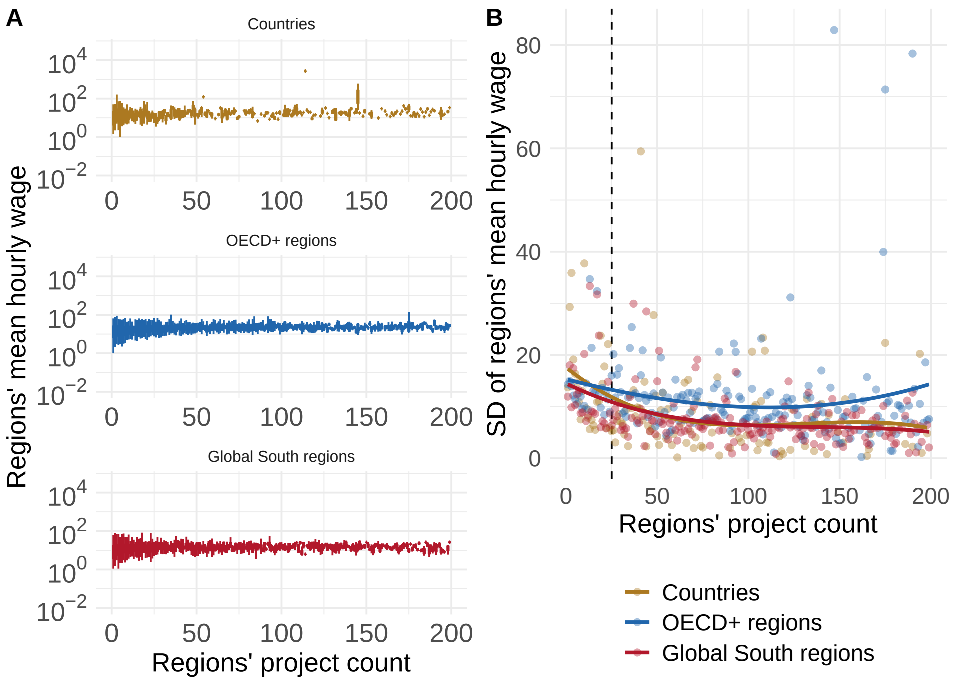

S Fig. 10 Variation of regional average wages in countries and regions. **(A)** Boxplots of the regional mean wages by project count. **(B)** Standard deviation of the mean hourly wages by project count. The average wages have sizeable dispersion in regions with very few observations, but they convert quickly to stable values in regions with a project count of 25 or more.

S 5.2 Explanatory features

After having transformed the output feature and identified an appropriate cut-off for the observations to be considered in the wage regressions, we consider the statistical relevance of different explanatory features. To do so, we have compared the adjusted R^2 of models with different collections of explanatory features (an example see Table 16 for different feature collections in OECD+ regional project count model) to identify the contribution of relevant control variables in explaining online labour project count and wages. We choose variables from a comprehensive list of features, which all represent typical variables used in geographical analyses of the platform economy.

Similarly, relevant features are considered for the other regression models on project count and

S Tab. 16 Relevant control variables from a comprehensive list of features are compared for their contribution in explaining the variance of the project count in OECD+ regions.

	OECD+ region project count (ihs-transformed)					
	(1)	(2)	(3)	(4)	(5)	(6)
Population, count	**1.17*****	**1.26*****	**1.19*****	**1.18*****	**1.47*****	**1.47*****
(log.)	(0.03)	(0.03)	(0.03)	(0.03)	(0.1)	(0.11)
Broadband Internet		**0.06*****	**0.04*****	**0.04*****	**0.04*****	**0.05*****
Household share		(0.002)	(0.002)	(0.002)	(0.003)	(0.003)
Tertiary education			**0.04*****	**0.04*****	**0.04*****	**0.04*****
Pop. share			(0.003)	(0.003)	(0.004)	(0.004)
Country capital				**0.7*****	**0.72*****	**0.78*****
(yes = 1/no = 0)				(0.12)	(0.12)	(0.13)
GDP per capita					**-0.27*****	**-0.21***
(log.)					(0.10)	(0.11)
ICT gross value added						-0.07
(log.)						(0.05)
Constant	−12.64***	−18.36***	−17.16***	−16.94***	−18.37***	−18.96***
	(0.50)	(0.42)	(0.42)	(0.42)	(0.68)	(0.79)
Observations	2.384	2.384	2.384	2.384	2.384	2.384
R^2	0.33	0.58	0.61	0.61	0.61	0.61
Adjusted R^2	0.33	0.58	0.60	0.61	0.61	0.61

Note: *p<0.1; **p<0.05; ***p<0.01

mean wages. To assure comparability across models for the interpretation of the results, we choose our final set of features (model 6) according to a list variables reflecting conceptualisations of major economic, human capital, and infrastructure variables, which are commonly used in analyses of the spatial distribution of online platform contributions (see section S 3) and which are available across all regional groups. We want to emphasise that the final choice of explanatory features is driven by theoretical considerations, not by extensive statistical feature selection. However, thanks to comparing different models, we could reduce the set of available explanatory features (see Table 8) to the most parsimonious collection, capturing an empirical measure of population, education, income per capita, and internet connectivity in all models. The country- and OECD+ models also contain a measure of the regional IT specialisation of the economy, the country model includes a measure of the price level and English language, and the OECD+ and Global South models contain an indicator variable for the capital region.

S 5.3 Model specification

In a final step, we choose the final model specification. First, we compare the chosen ihs-transformed OLS model with alternative model specifications of a log-transformed model without zero values and a negative binomial model, as shown in Table 17.

There are no substantial differences with regards to the inclusion or exclusion of the zero-count regions (comparison between models 1. and 3.). We therefore stick to our choice of assigning

S Tab. 17 Comparison of model specifications in explaining OECD+ regional project count.

	OECD+ region project count			
Transformation	ihs-transformed		log-transformed[a]	simple count
Model	OLS	OLS Country-year FE	OLS	Neg.Binomial
	(1)	(2)	(3)	(4)
Population, count	**1.47*****	**0.85*****	**1.19*****	**1.50*****
(log.)	(0.11)	(0.09)	(0.10)	(0.09)
Education	**0.04*****	0.004	**0.04*****	**0.04*****
Pop. share with tertiary education	(0.004)	(0.01)	(0.003)	(0.003)
GDP per capita	**-0.21***	**-0.33*****	**-0.21****	**-0.52*****
(log.)	(0.11)	(0.1)	(0.11)	(0.09)
Household share with broadband	**0.05*****	**0.03*****	**0.04*****	**0.04*****
	(0.003)	(0.004)	(0.003)	(0.002)
ICT gross value added	-0.07	**0.56*****	0.01	0.03
(log.)	(0.05)	(0.05)	(0.05)	(0.04)
Region holds country capital	**0.78*****	0.13	**0.67*****	**0.94*****
(yes = 1/no = 0)	(0.13)	(0.10)	(0.11)	(0.11)
Constant	-18.96^{***}		-14.85^{***}	-15.81^{***}
	(0.80)		(0.77)	(0.70)
Observations	2.384	2.384	2.157	2.384
R^2	0.61	0.70	0.57	
Adjusted R^2	0.61	0.67	0.57	

Note: *p<0.1; **p<0.05; ***p<0.01

[a] The log-transformed model lacks 227 zero valued observations.

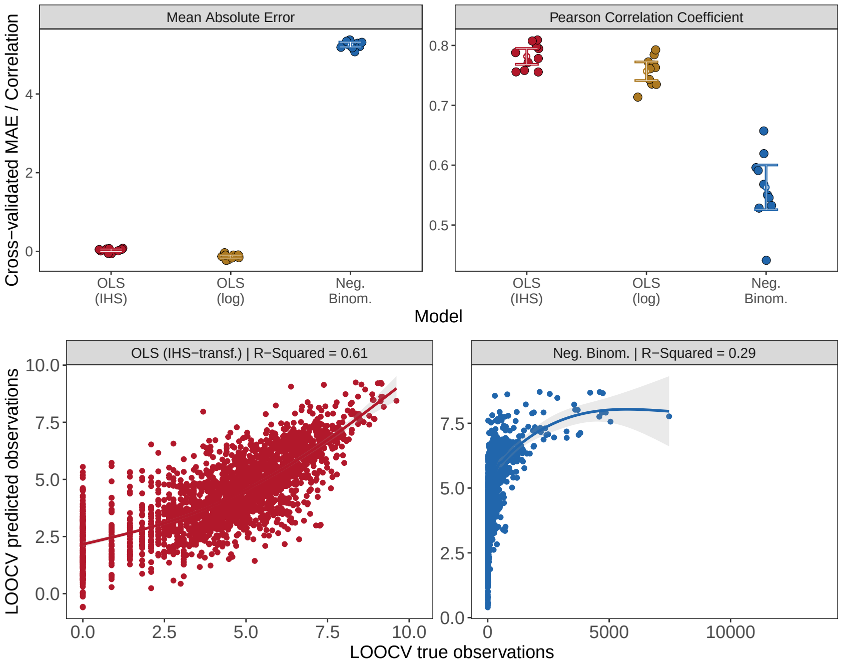

S Fig. 11 Model comparison in terms of their out-of-sample prediction accuracy. Upper panel: Mean Absolute Error (MAE) and Pearson correlation coefficient ρ of 10-fold cross validated samples in comparison to test data. Lower panel: Comparison of Leave-one-out cross-validated samples predictions and observed values. The ihs-transformed ordinary least squares (OLS) model shows a higher prediction accuracy than the negative binomial model,.

zero values to regions in which no projects were detected. Moreover, we observe that the negative binomial model (4.) yields very similar results compared to the linear models. However, the negative binomial model is less accurate in terms of cross-validated out-of-sample prediction accuracy (Fig. 11, upper panel).

By applying leave-one-out cross validation, we control for the effects of outliers and detect that the negative binomial models fail to accurately predict extreme contribution values (Fig. 11, lower panel). We therefore conclude that the ihs-transformed OLS model is best suited to describe the regional project count by socio-economic local factors. After further testing, this conclusion likewise applies to the other five regression models relating project count and hourly wages to to local factors on the country- and regional level.

As our models deal with different hierarchical levels of data, e.g., regions nested in countries, multi-level effects need to be considered. We test and apply random and fixed effect multi-level models to account for the variability of outcomes within and across country or year groups. The consideration of yearly fixed effects is, in any case, advisable, as our project and wage data has been derived from separate annual samples (see section S 4.1). Figure 12 illustrates, with the example of OECD+ regions, that average project counts vary significantly across countries and years.

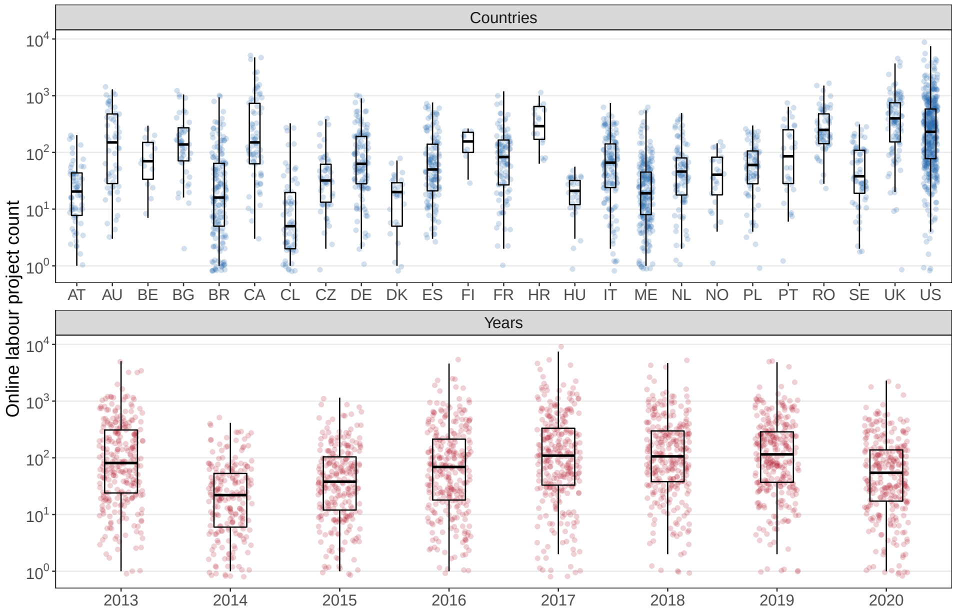

S Fig. 12 Country-level (upper panel) and yearly (lower panel) boxplots of online labour project count in OECD+ regions: project counts vary significantly across countries and years, indicating the relevance of level effects to be considered in regression modelling.

In addition, for our wage regressions, the comparison of the relationship between wage and central characteristics, such as broadband connectivity, advises the use of random effect controls.

Besides statistical reasons, there are theoretical considerations that suggest the application of random effect controls for wage but not for project count regression, as illustrated according to the example of Figure 13.

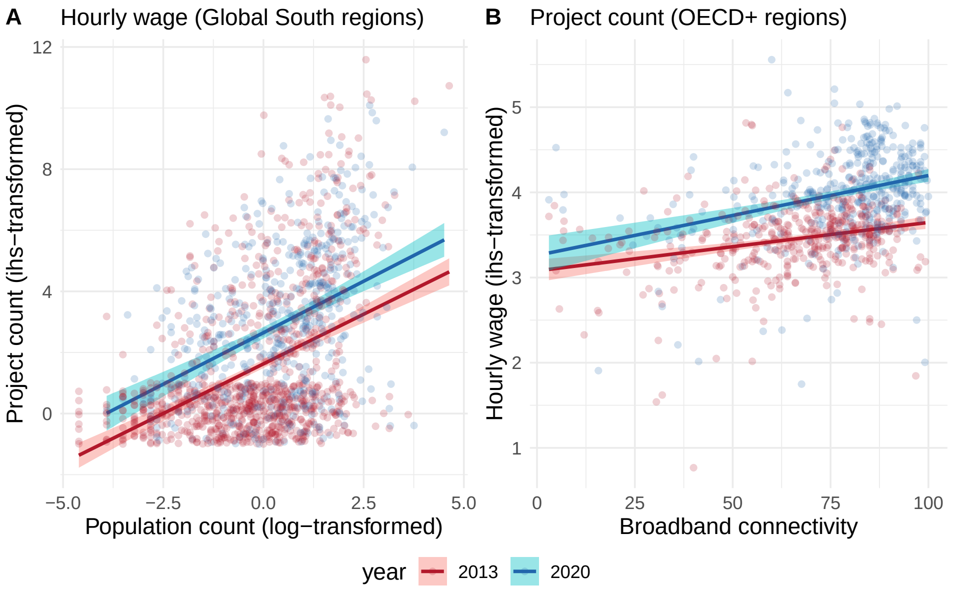

S Fig. 13 Indications for fixed and random level effects: For Global South regions, the relationship between population and project count show differences in levels across years (A), in the case of OECD+ regions, the relationship between broadband connectivity and wages shows different slopes across time (B).

When explaining regional project count, level fixed effects matter. For example, as the market of remote platform work matures over time, more projects will be attributed to the same population sized region in 2020 than it was the case in 2013. However, the slope of the positive relationship between head count and project count does not change (A). In the case of wages, however, different dynamics unfold over the course of seven years. In 2020, one additional unit (percentage point) in broadband access, contributes more to rising wages than in 2013. This could be explained by the nature of well-paid jobs, as these have become more data heavy from 2013 to 2020 and therefore increasingly require better internet infrastructure; the positive slope between broadband connectivity and wages tilted upwards moving from 2013 to 2020.

In addition to fixed effects, random effect models require specific considerations [43] that need to be accounted for, as we address in the following. First, random effects are "data hungry": they require — as a rule of thumb — at least five levels (groups) for a random intercept term to achieve robust estimates of variance. This condition is satisfied in our case. Secondly, random effects models can be unstable if sample sizes across groups are unevenly distributed, e. g., some groups are much larger than others. The within effects of the larger sample size groups can skew the direction of the overall regression coefficients. However, for the coefficients in our example, we observe a similar directionality across all groups, e. g., the relationship between broadband

and wages did change over time, but it remains clearly positive. Thirdly, an incorrect 'level' specification of a random effects model can ultimately lead to pseudo-replication and inflated Type I error rates. We acknowledge this possibility by applying F-tests, which provide a check of model hierarchy using residual degrees of freedom. Lastly, as with any inferential model, the issue of endogeneity arises. Just like for the individual level, nested within the groups, we have to argue that no hidden confounder on the group level jeopardises a consistent estimation by deterring a zero covariance. While one can, for theoretical reasons, never entirely exclude the possibility of endogeneity, we argue for the use of random effects in light of a trade-off: The F-Test indicates that the model requires the control for level effects. Furthermore, the Hausmann-Test shows us that a random effects setting leads to a better reduction of error term variance than a fixed effects setting[15]. In this situation, we could either not include level effects to avoid potential confounder on the group level or we include level effects, in this case random effects, to account for the different residual variance distribution across groups. We decide on the second alternative.

Accordingly, the adjusted R^2 of our optimised model increases, once adjusting for country and year fixed effects, shown in model (2) of Table 17. After performing F-tests for the existence of level effects and Hausman tests [44] for the comparison of fixed versus random effects, we conclude that, for OECD+ and GDL regions, both, wage regressions (random effects model) and project regressions (fixed effects model), require level effects for countries and years, as shown in the model summary in Figure 2A of the main text.

S 6 Additional analyses

S 6.1 Robustness of regression results to data imputation

As outlined in section S 4.3, we have imputed missing data points in the regional data sets from the World Bank, OECD, and GDL in order to reduce the number of observations that need to be dropped in the geographical regression models. Unfortunately, 25 % of the values in the country data set, 30 % of the values in the OECD+ regional data set, and 41 % of the values in the GDL data set are missing. This results in 60 % of the rows in the country data set, 86 % of the rows in the OECD+ data set, and 85 % of the rows in the GDL data set having at least one missing variable.

This would imply that a majority of data points could not be used for the geographical regression models, which require complete data. Therefore, a reasonable imputation of missing data points — one that does not systematically distort the data — reduces the number of rows that have to be dropped from the tables used in the regression models and thus leads to more accurate parameter estimations based on more observations. In our case, there are three cases of missing that

[15]GDL regions: $F = 1.6342^{***}, \chi^2 = 108.16^{***}$, OECD+ regions: $F = 2.64^{***}, \chi^2 = 15.07^{**}$

need to be distinguished for possible imputation. First, individual observations of one region in one year are missing. In this case, we replaced the missing value with the value from the previous year. Secondly, all observations of one region are missing, but data from other regions in the same country are present. In that case, we have imputed with the unconditional average of the other regions. Such imputation is, however, only possible in cases of ratios and similar variables. For example, replacing the share of people with tertiary education or the share of households with broadband access with the country average is valid, but it would not be feasible to replace missing values of the total gross value added in ICTs in a region as such a value would be dependent on a region's population and other factors. Thirdly, if all values of a variable are missing in a country, imputation is not possible.

In summary, imputing missing values increases the number of rows with complete observations to 68 % in the country-level data (1,1136 of 1,168 country-year combinations), 43 % in the OECD+ region data (2,384 of 5,505 region-year combinations), and 31 % in the GDL data set (2,074 of 6,654 region-year combinations). Comparing Table 2A from the main text with Table 18 in this section, which presents regression models on the data without any imputation, shows that the results are not substantially affected by the imputation. In fact, many of the parameter estimates are almost identical and the direction of the effect is the same in all cases. However, in some cases, the results are not statistically significant, as standard deviations are larger.

S Tab. 18 Regression models from Fig. 2A of the main text without imputed missing values. The overall results (direction, magnitude) have not been affected by the imputation, but some coefficients are not statistically significant in the non-imputed case presented here, due to larger standard deviations.

Dependent variable:	Yearly online labour project count			Online labour avg. wage per hour		
Level:	Countries	Sub-national regions		Countries	Sub-national regions	
Geography:	Global	OECD+	Global South	Global	OECD+	Global South
Model:	(1)	(2)	(3)	(4)	(5)	(6)
Population	**0.97*****	**0.69*****	**0.64*****	**0.03*****	0.03	**0.09*****
Population, total (log scale)	(0.03)	(0.16)	(0.05)	(0.01)	(0.07)	(0.03)
Education	**0.07*****	0.003	**0.08***	**0.01*****	**0.01*****	**0.04****
Model (1), (4): share of pop. with secondary education	(0.004)	(0.01)	(0.04)	(0.002)	(0.002)	(0.02)
Model (2), (5): share of pop. with tertiary education						
Model (3), (6): avg. years of education						
Income per capita	−**0.02*****	−0.27	**0.05***	0.002	−0.07	**0.04*****
Model (1), (4): GDP per capita (in 1,000 $)	(0.004)	(0.17)	(0.03)	(0.001)	(0.08)	(0.01)
Model (2), (5): GDP per capita (2015 PPP $, log scale)						
Model (3), (6): Gross National Income p. c. (2011 PPP $)						
Internet connectivity	**0.03*****	**0.03*****	**0.01***	**0.01*****	0.002	**0.01*****
Model (1), (4): fixed broadband subscriptions per 100 people	(0.01)	(0.01)	(0.01)	(0.003)	(0.002)	(0.002)
Model (2), (5): share of HHs with internet broadband access						
Model (3), (6): share of HHs with internet access						
IT specialisation of the economy	**0.26*****	**0.62*****		−0.01	**0.07***	
Model (1), (4): ICT share of all service exports (log scale)	(0.06)	(0.09)		(0.02)	(0.04)	
Model (2), (5): Gross value added in ICT (2015 PPP $, log)						
English language	**0.78*****			−**0.09****		
Indicator: English is official language	(0.14)			(0.04)		
Price level	−**0.36*****			−**0.07***		
PPP conversion factor (per 1,000 int. $)	(0.11)			(0.04)		
Capital region		0.10	**1.75*****		−0.06	−**0.15***
Indicator: region holds country capital		(0.16)	(0.15)		(0.06)	(0.08)
Constant					3.26***	2.37***
					(0.52)	(0.17)
Observations	661	813	944	444	556	104
Fixed / Random Effects	Yearly FE	Country-year Fixed Effects		Yearly FE	Country-year Random Effects	
R^2	0.70	0.70	0.44	0.35	0.83	0.51
Adjusted R^2	0.69	0.67	0.37	0.33	0.83	0.49
Note:						*p<0.1; **p<0.05; ***p<0.01

S 6.2 Analysis of model residuals

In Figure 2B of the main text, we provide a graphical depiction of the variance reduction achieved by the spatial regression models (1) and (4). We find that a large share of the global differences in online labour market activity and hourly wages can be explained with relatively a small set of economic, infrastructure and human capital variables. Here, we want to provide more aspects related to this finding. Figure 14 shows the distribution of online labour market activity (upper panel) and hourly wages (lower panel) between countries on an ihs-transformed scale (red) and the model residuals of three types of models. The first set of models (blue) explains global differences with price differences alone (Price level - PPP conversion factor per 1,000 int. $), the second set of models (yellow) relates the outcome variables to differences in secondary education (share of population with secondary education), and the third set (green) represent the optimised models shown in Table 2A of the main text.

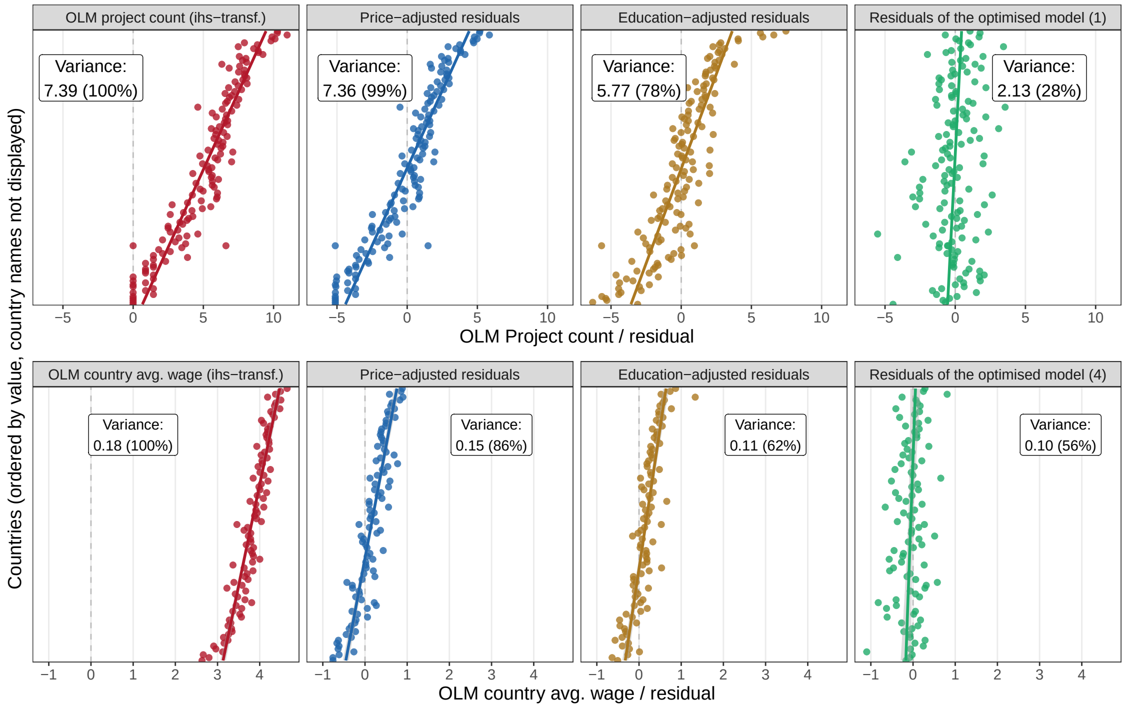

S Fig. 14 Distribution of online labour (OLM) project count and avg. hourly wages per country (left) and residual plot of three types of models: price-adjusted residuals (blue), education-adjusted residuals (yellow), and residuals of the optimised models (green). Differences in global human capital distribution explain more of the overall variation than price levels alone. The parsimonious complete models explain a large share of the overall variation.

As highlighted in the main text, price differences alone do not explain much of the global variation, neither in terms of online labour market activity ($R^2 = 1\,\%$) nor in terms of hourly wages ($R^2 = 14\,\%$). This observation suggests that jobs in the online labour market do not just follow differences in prices and living costs. In that sense, the online labour market is not a 'level-playing field' [45] or a 'flat world' [46]. Geographical frictions prevent jobs from going to

those places that could offer labour for the lowest wages. Instead, as we see in the third panel, differences in secondary education do explain a larger share of the overall distribution in online labour project count ($R^2 = 22\,\%$) and hourly wages ($R^2 = 38\,\%$). Differences in the global human capital distribution, hence, play a more substantial role in driving the polarisation of platform work than differences in price levels. The fourth panel shows the optimised models, which are able to explain a major share of the overall variation in project count ($R^2 = 72\,\%$) and hourly wages ($R^2 = 44\,\%$).

S 6.3 Sub-national granularity

Online labour market activity is highly concentrated in a limited number of places. This spatial concentration becomes apparent if the data is mapped to sub-national geographical entities. Here, we illustrate this pattern on the case of Europe, where the NUTS statistical regions allow to associate online labour data relatively easily to sub-national regions of comparable size. To do so, we use geographical information systems packages in `R` to match the geocoded online projects to geographical boundary files provided by Eurostat.[16] Figure 15 shows the online labour project count from the 2020 data sample on the NUTS 0 level (countries), NUTS 1 level (corresponding to large sub-national regions, e. g. federal states in Germany), NUTS 2 level (corresponding to medium-sized regions, e. g. Government regions in Germany), and NUTS 3 level (corresponding to small regions, e. g. districts in Germany).

It becomes obvious that country-level data does not reveal the fine-grained spatial distribution of online in Europe. The NUTS 1 and NUTS 2 aggregations make some of the sub-national variation visible, but only the smallest regional aggregation reveals the concentration in urban areas to a full degree. In many countries, most activity takes place in urban centres. However, in South-East Europe, where wage levels tend to be lower than in West Europe, online labour market activity is distributed more evenly across space.

Overall, online labour market activity in Europe shows a similar distribution across all levels of spatial aggregation, highlighted in Figure 16. Independently of the granularity chosen, we observe that most of the total project count is concentrated in a few countries, large regions, medium-sized regions, or small regions. The more fine-grained the granularity, the more heavy-tailed the distribution. Spatial polarisation in the online labour market is, thus, not only relevant on the country level, but equally on smaller geographical entities. It is only this finer granularity that reveals the inequalities between urban and rural areas, which seem to be a global feature of digital platform work.

[16] `http://ec.europa.eu/eurostat/web/gisco/geodata/reference-data/administrative-units-statistical-units/nuts`

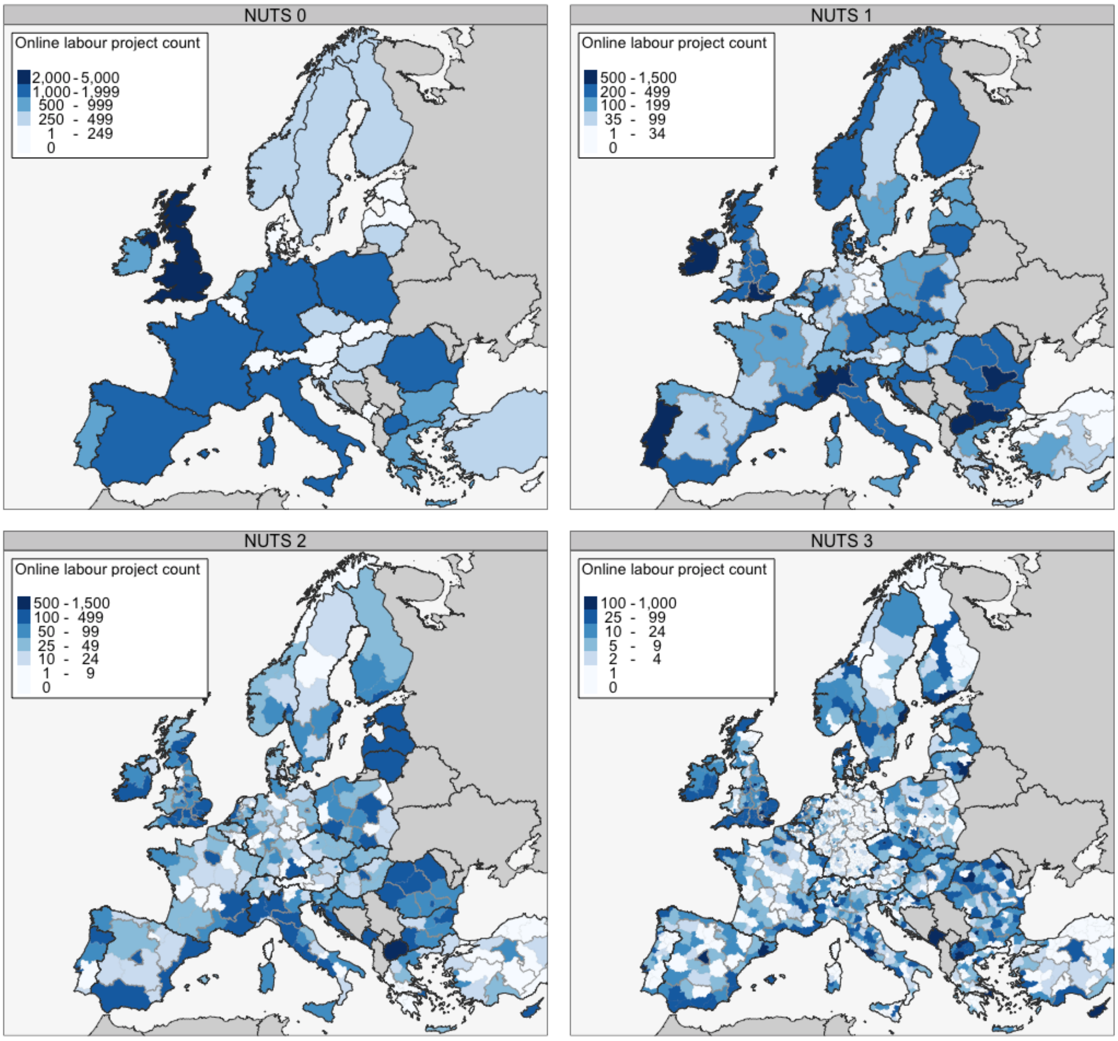

S Fig. 15 Online labour market activity in Europe in 2020 in different statistical aggregations (NUTS 0 – NUTS 3). The maps show that online labour market activity is concentrated in urban centres within Europe. This level of urban-rural spatial concentration can not be detected if the data is aggregated on the country level only.

S 6.4 Wage distribution across countries and occupations

In contrast to traditional labour markets, which are characterised by average wage spreads between countries that are often more important those between occupations within a country [47, 48], we observe that in the online labour market, what you do is more important than where you do it. Figure 17 shows the spread of wages between countries (upper panel) and occupations (lower panel). Each boxplot covers all the yearly observations per country / occupation. Overall, the distribution of the average (mean) hourly wages is similar in both dimensions. The majority of average wages lies around $ 20, with the 5 % quantile being $ 8 for occupations and $ 9 for countries. The 95 % quantile is $ 46 for occupations and $ 38 for countries, but the spread within occupations over the years seems to be smaller than the spread within countries over time (length

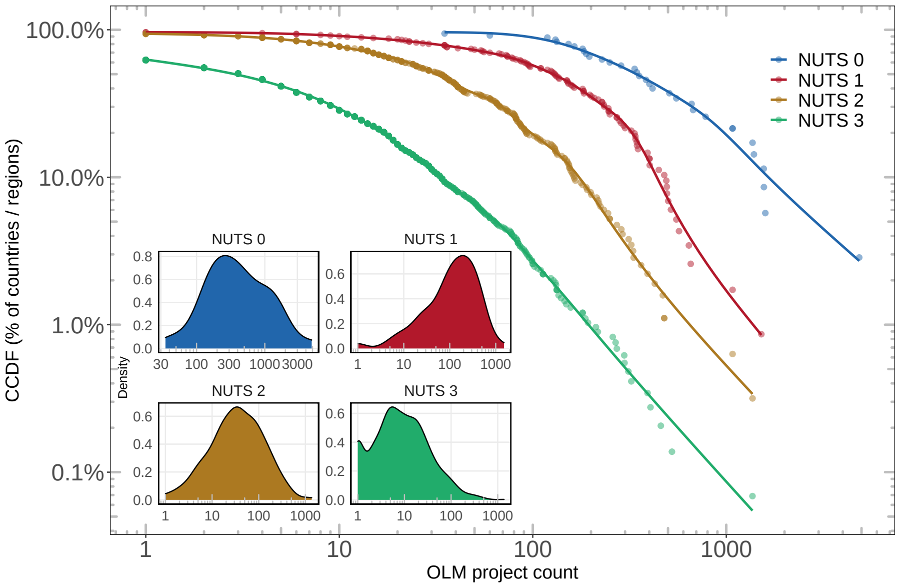

S Fig. 16 Distribution of online labour market activity in Europe in different statistical aggregations (NUTS 0 – NUTS 3). Main plot: complementary cumulative distribution function (CCDF) of regions (dots) on a log-log plot and smoothed trend lines. Inset: density plots. The spatial concentration is similarly distributed across all spatial levels, but becomes more right-skewed the finer the granularity.

of the boxplots).

From these observations, we infer that occupations, in other words types of activities that require particular skill combinations, form individual sub-markets in the overall global market. To some extent, platform workers can migrate between the sub-markets, but the different requirements and conditions within each sub-market prevent a free flow of labour between them.

S 6.5 Polarisation over time

How did the three dimensions of polarisation (differences between and within countries and differences between occupations) develop over time? Do we see convergence or divergence? The data we collected does not allow to compare overall project counts between years (because of the different collection approaches, see secction S 4), but we can use it compare the distribution of project counts and average wages between countries and regions, as well as between occupations. Figure 18 and Figure 19 illustrate how the polarisation developed along these two axes.

Figure 18A shows the number of participating countries and regions in the three data sets. Independent of the drop in 2014 and 2015 (which is due to the data collection approach used to gather data for these two years), we observe that the number of geographical entities did not grow substantially over time. A few countries and regions joined the online labour market, but overall the market had a global scope already in the first year of the observation period. Nonetheless, as shown in the statistically significant decreases of the Gini coefficients in Figure 18B, the spatial concentration between countries and regions decreased on a global level over time. Online labour

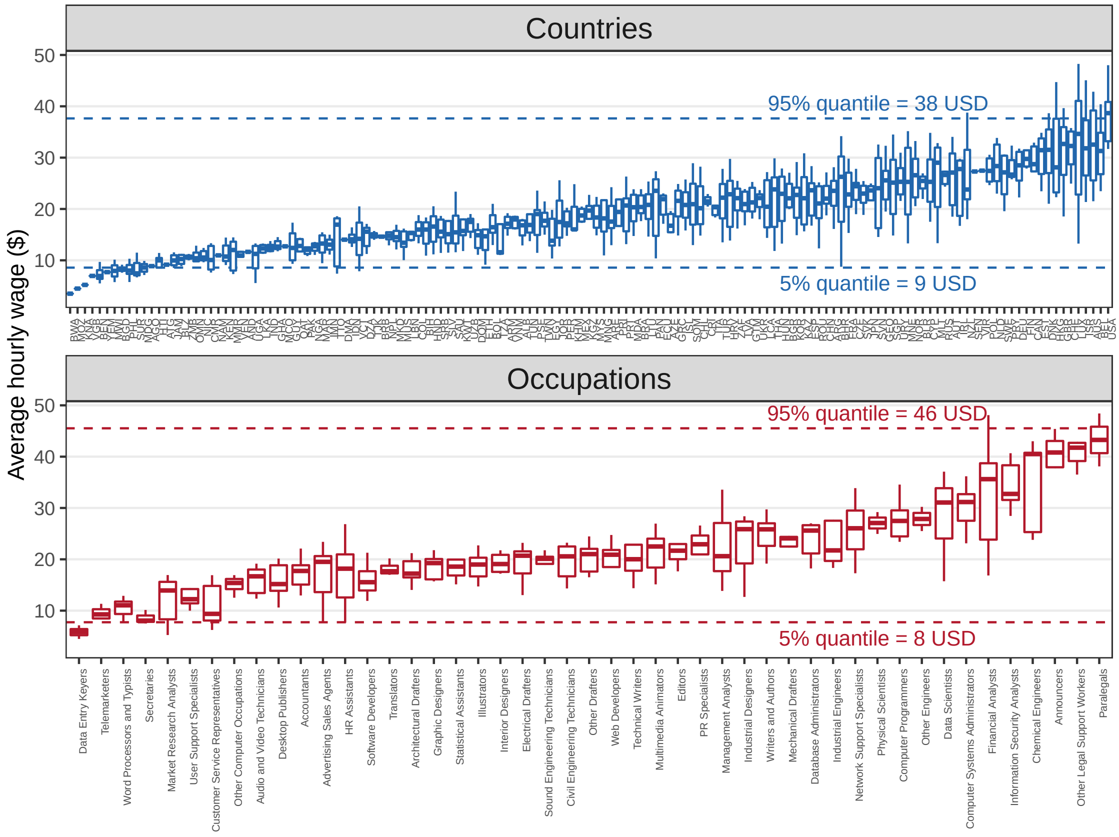

S Fig. 17 Average hourly wages ($) across countries (upper panel) and occupations (lower panel). The spread between occupations is larger than that between countries.

platforms were used by platform workers from a larger set of places more actively, reducing the overall high level of spatial inequality to some extent. However, the reduction in inequality was not equal everywhere. Compared to Global South regions that saw an average decrease in the Gini coefficient of 0.4 % points per year, the inequality between OECD+ regions (the regions that did accumulate the largest share of projects anyway), decreased by 1.5 % points per year, i. e. four times more. In other words, the global reduction in spatial polarisation in online labour market activity is largely due to a more equal participation among OECD+ regions and countries.

Figure 18C looks into the second polarisation dimension: spatial concentration within countries. The boxplots show the Gini coefficient within countries in the Global South (left) and OECD+ (right), considering only the non-capital regions of each country. The findings are similar to those of the between-country inequalities. The level of concentration is higher in Global South countries than in OECD+ countries and the decrease over time is lower in the Global South. However, the decrease is not statistically significant. Thus, the concentration within countries remained relatively stable over time. Panel D complements this observation in showing the share of projects conducted by capital regions in Global South countries (left) and OECD+ countries

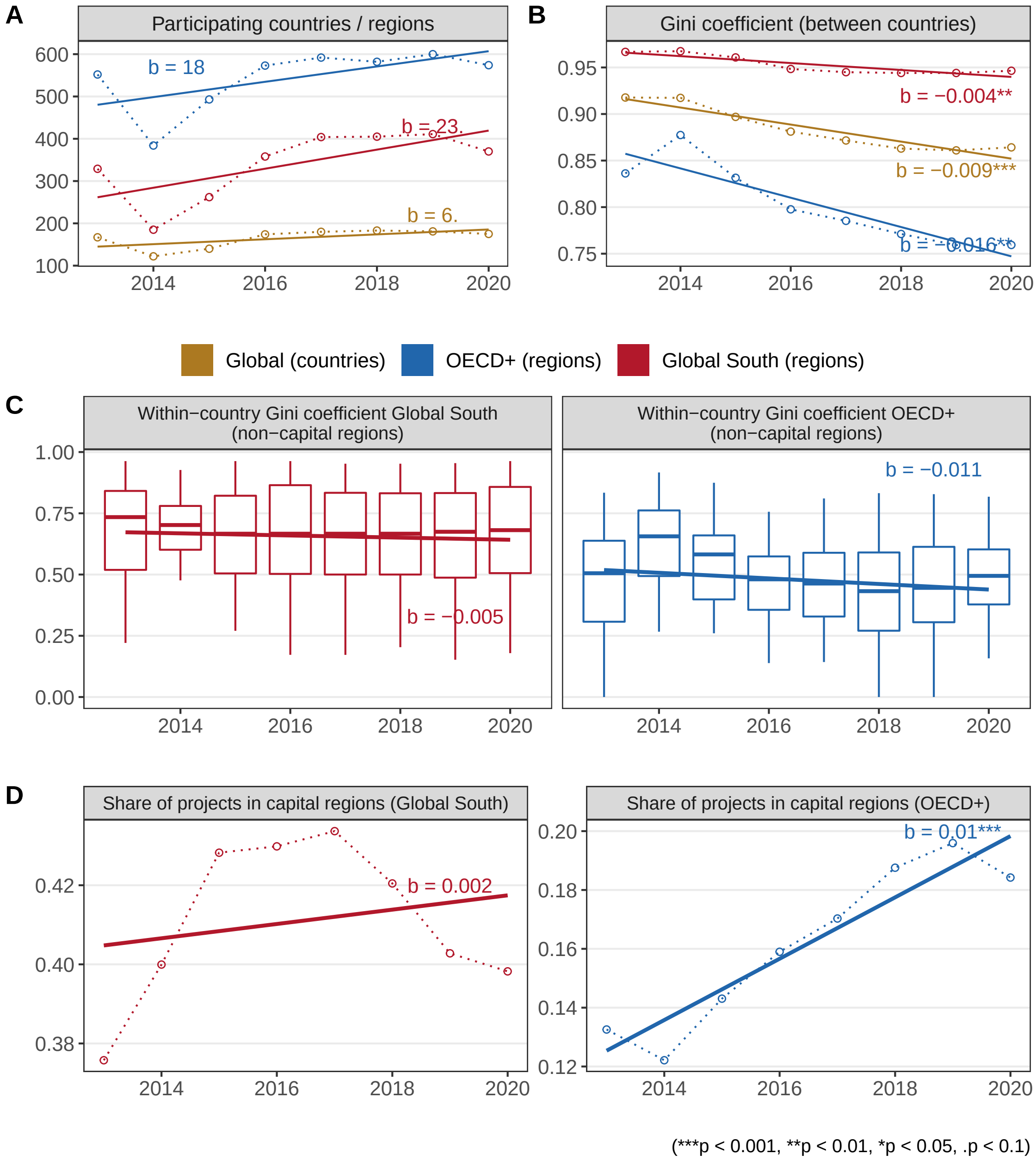

S Fig. 18 Polarisation over time (project count). **(A)** Number of countries / regions that participated in the online labour market with at least one project per year. **(B)** Gini coefficient of the total project count per year between countries / regions. **(C)** Boxplot of within-country Gini coefficients in non-capital regions in Global South (left) and OECD+ (right) countries over time. **(D)** Share of projects conducted by capital regions in Global South (left) and OECD+ (right) countries over time. While spatial concentration decreased between countries and regions on a global scale, polarisation within non-capital regions in countries remained constant and the share of metropolitan (capital) regions increased.

(right). The capital share in the Global South shows an inverse U-shaped pattern, probably due to a higher diffusion of online labour platforms in non-capital regions of these countries in the most recent years of the observation period. However, the capital share is with around 40 % of all projects being done in the capital regions of Global South countries, overall, very high. In contrast, the share of projects conducted by capital regions in OECD+ countries started at a lower level

(13 %) but grew constantly by around 1 % point on average over the observation period, having reached a share of around 19 % by 2020.

These findings suggest the following interpretation. Online labour markets were and are global in scope. They are, in theory, open to participation from everywhere. However, as shown by the regression models in the main text, this participation is conditioned by the local economy, human capital and infrastructure. Therefore, we observe substantial differences in the level of spatial concentration between regional groups. Within countries, the polarisation along the urban-rural dimension plays out fully, in both Global South countries and high income countries. As in the overall economy, the booming metropolitan areas become more and more important, leaving 'the broken provincial cities of the past' [49] behind.

A similar finding is made when looking into the development of hourly wages over time. Figure 19A shows the wage development in of countries (left) and occupations (right) in three wage groups: the lowest 33 % quantile (bottom), the middle quantile, and the top quantile, based on the wage level in 2013 (countries and occupations that joined the online labour market in later years are not displayed here to ease interpretation of the results). The positive observation is that wages grew throughout all wage groups in both the country and occupation data sets over time. However, it is not clear whether these growing wages are only inflation-correcting or actually yielding higher real wages. If we compare the wage development between the groups, it becomes obvious that the top third saw the highest growth rates, in particular in the occupation data. In contrast to the bottom group of occupations, which saw an average wage growth of $ 1.25 per year, wages grew by $ 1.67 per year in the top group. Thus, the wage differences between the types of jobs that promise the highest income potential for platform workers and those that offered lowest wages became larger over time; an observation that emphasises the relevance of occupations as a main dimension of polarisation in the online labour market. Similarly, the wages in the lowest country quantile did not grow significantly over time ($ 0.55 per year on average), while those in higher wage countries did increase almost twice as as high ($ 1.19 and $ 1.26, respectively).

Looking into the wage development in capital and non-capital regions in Figure 19B shows the differences between Global South (left) and OECD+ countries (right). The wages are, unsurprisingly, lower in Global South regions than in OECD+ regions, and the average wage increases also differ between the groups. Overall, the wages grew by similar rates in both capital and non-capital regions. In other words, the differences in wage levels are not reduced over time. Platform workers in metropolitan areas, particularly in the Global South, earn more than their counterparts in rural areas and this difference is persistent over time. Only in OECD+ regions, where wage differences have been less pronounced, we see a catch-up of non-captial regions in the most recent years.

Comparing wages across occupations and regions underlines the polarisation dimensions discussed in the main text. The online labour market mirrors the polarisation of the increasingly

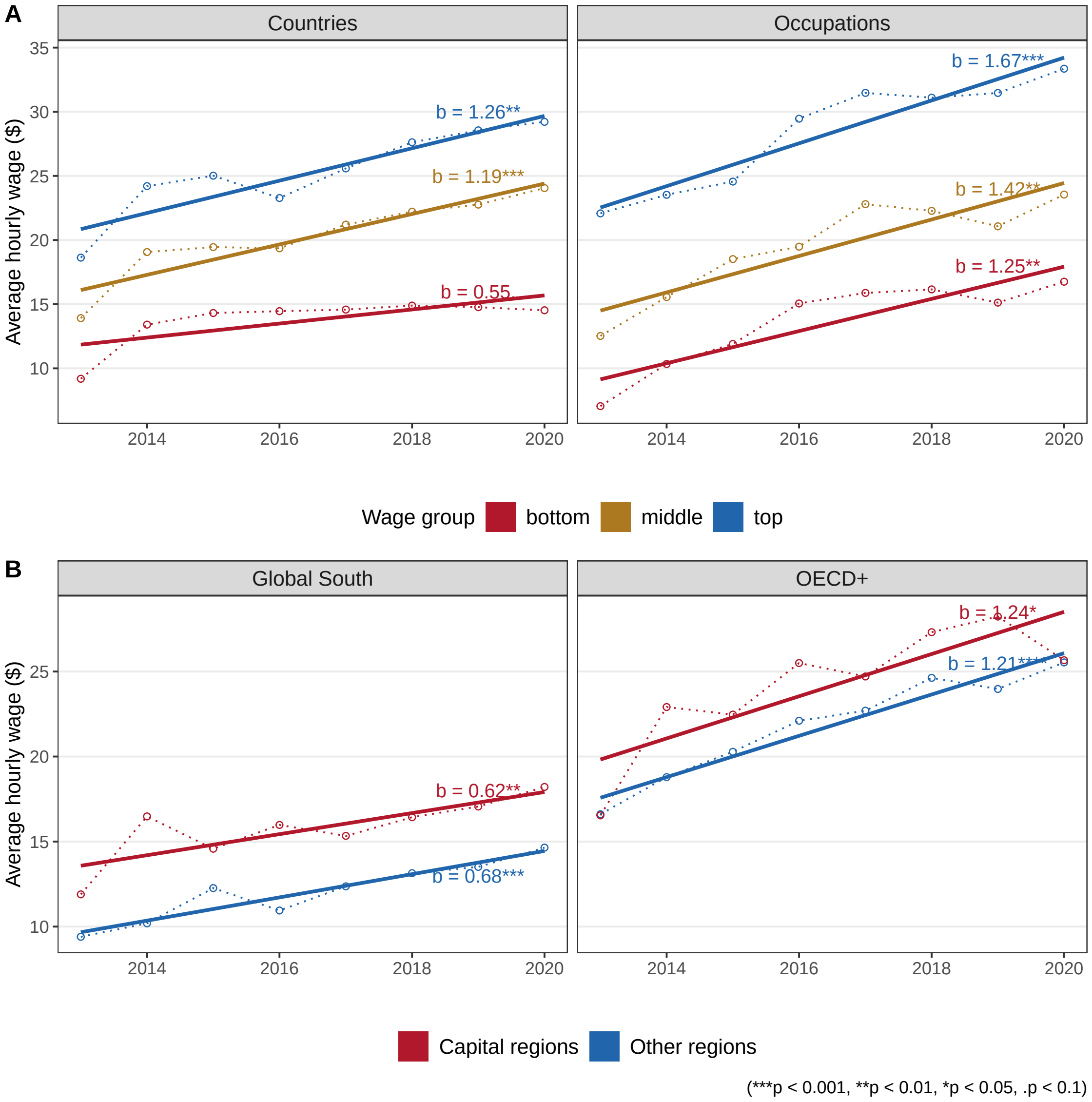

S Fig. 19 Wage development over time in wage groups (based on 33 % quantiles in 2013 data) and capital vs. non-capital regions. (A) Wages grew over time in all groups, but wage growth was largest in the top-wage countries (left) and top-wage occupations (right). The wages in the bottom country group did not grow substantially. (B) Wages grew in capital and non-capital regions, both in Global South (left) and OECD+ (right), but the spread between metropolitan areas and other regions remained constant over time.

globally connected labour markets and the polarisation along the occupation- or skill dimension increasingly dominates differences across space. The type of job a platform worker can offer digitally shapes the opportunities in the online labour market, just as the job type a person works in determines the individual fate either to obtain a skill-based premium in the global 'war for talent' [50–52] or to fight for a decent living in the 'global auction' [47].

References

[1] Debra Howcroft and Birgitta Bergvall-Kåreborn. A Typology of Crowdwork Platforms. *Work, Employment and Society*, 33(1):21–38, 2019.

[2] Michele Cantarella and Chiara Strozzi. Workers in the crowd: the labour market impact of the online platform economy. 2019.

[3] Yili Hong and Paul A Pavlou. Is the world truly'flat'? empirical evidence from online labor markets. *Empirical Evidence from Online Labor Markets (October 1, 2014). Fox School of Business Research Paper*, (15-045), 2014.

[4] John Horton. Online Labor Markets. In Amin Saberi, editor, *Internet and Network Economics*, Lecture Notes in Computer Science, pages 515–522. Springer Berlin Heidelberg, 2010. ISBN 978-3-642-17572-5.

[5] John Horton, William R. Kerr, and Christopher Stanton. Digital labor markets and global talent flows. Working Paper 23398, National Bureau of Economic Research, Cambridge MA, 2017.

[6] Ajay Agrawal, John Horton, Nicola Lacetera, and Elizabeth Lyons. Digitization and the Contract Labor Market: A Research Agenda. *Economic Analysis of the Digital Economy*, pages 219–250, 2015.

[7] Ajay Agrawal, Nicola Lacetera, and Elizabeth Lyons. Does standardized information in online markets disproportionately benefit job applicants from less developed countries? *Journal of International Economics*, 103(C):1–12, 2016. ISSN 0022-1996. doi: 10.1016/j.jinteco.2016.08.003.

[8] Katharine A. Anderson. Skill networks and measures of complex human capital. *Proceedings of the National Academy of Sciences*, page 201706597, 2017. ISSN 0027-8424. doi: 10.1073/pnas.1706597114.

[9] Florian A Schmidt. Digital labour markets in the platform economy: Mapping the political challenges of crowd work and gig work. *Bonn: Friedrich-Ebert-Stiftung*, 2017.

[10] Mark Graham and Mohammad Amir Anwar. The global gig economy: Towards a planetary labour market? *First Monday*, 24(4), 2019.

[11] Yili Hong and Paul Pavlou. On Buyer Selection of Service Providers in Online Outsourcing Platforms for IT Services. *Information Systems Research*, 28, April 2017. doi: 10.1287/isre.2017.0709.

[12] Fabian Braesemann, Vili Lehdonvirta, and Otto Kässi. ICTs and the Urban-Rural Divide: Can Online Labour Platforms Bridge the Gap? SSRN Scholarly Paper ID 3271972, Social Science Research Network, Rochester, NY, 2018.

[13] Uma Rani and Marianne Furrer. On-Demand Digital Economy: Can Experience Ensure Work and Income Security for Microtask Workers? *Jahrbücher für Nationalökonomie und Statistik*, 239(3):565–597, 2019. ISSN 0021-4027. doi: 10.1515/jbnst-2018-0019.

[14] Vili Lehdonvirta, Otto Kässi, Isis Hjorth, Helena Barnard, and Mark Graham. The Global Platform Economy: A New Offshoring Institution Enabling Emerging-Economy Microproviders. *Journal of Management*, 45(2):567–599, 2019.

[15] Siou Chew Kuek, Cecilia Paradi-Guilford, Toks Fayomi, Saori Imaizumi, Panos Ipeirotis, Patricia Pina, and Manpreet Singh. The Global Opportunity in Online Outsourcing. Working Paper ACS14228, World Bank Group, Washington D.C., 2015. URL `http://documents.worldbank.org/curated/en/138371468000900555/The-global-opportunity-in-online-outsourcing`.

[16] Ajay Agrawal, John Horton, Nicola Lacetera, and Elizabeth Lyons. Digitization and the contract labor market: A research agenda. In *Economic analysis of the digital economy*, pages 219–250. University of Chicago Press, 2015.

[17] Yili Hong and Paul A Pavlou. On buyer selection of service providers in online outsourcing platforms for it services. *Information Systems Research*, 28(3):547–562, 2017.

[18] Niels Beerepoot and Bart Lambregts. Competition in online job marketplaces: towards a global labour market for outsourcing services? *Global Networks*, 15(2):236–255, 2015.

[19] Ajay Agrawal, Nicola Lacetera, and Elizabeth Lyons. Does standardized information in online markets disproportionately benefit job applicants from less developed countries? *Journal of international Economics*, 103:1–12, 2016.

[20] Ejaz Ghani, William R Kerr, and Christopher Stanton. Diasporas and outsourcing: evidence from odesk and india. *Management Science*, 60(7):1677–1697, 2014.

[21] Uma Rani and Marianne Furrer. On-demand digital economy: Can experience ensure work and income security for microtask workers? *Jahrbücher für Nationalökonomie und Statistik*, 239(3):565–597, 2019.

[22] Fabian Braesemann, Vili Lehdonvirta, and Otto Kässi. Icts and the urban-rural divide: can online labour platforms bridge the gap? *Information, Communication & Society*, pages 1–21, 2020.

[23] Kathrin Borchert, Matthias Hirth, Michael E Kummer, Ulrich Laitenberger, Olga Slivko, and Steffen Viete. Unemployment and online labor. *ZEW-Centre for European Economic Research Discussion Paper*, (18-023), 2018.

[24] Martin Lukac and André Grow. Reputation systems and recruitment in online labor markets: insights from an agent-based model. *Journal of Computational Social Science*, pages 1–23, 2020.

[25] Vili Lehdonvirta, Otto Kässi, Isis Hjorth, Helena Barnard, and Mark Graham. The global platform economy: A new offshoring institution enabling emerging-economy microproviders. *Journal of Management*, 45(2):567–599, 2019.

[26] Alex J Wood, Mark Graham, Vili Lehdonvirta, and Isis Hjorth. Good gig, bad gig: Autonomy and algorithmic control in the global gig economy. *Work, Employment and Society*, 33(1): 56–75, 2019.

[27] Martin Lukac. Two worlds of online labour markets: Exploring segmentation using finite mixture models and a network of skill co-occurrence. 2021.

[28] Amanda Pallais. Inefficient hiring in entry-level labor markets. *American Economic Review*, 104(11):3565–99, 2014.

[29] John J Horton. The effects of algorithmic labor market recommendations: Evidence from a field experiment. *Journal of Labor Economics*, 35(2):345–385, 2017.

[30] Christopher T Stanton and Catherine Thomas. Landing the first job: The value of intermediaries in online hiring. *The Review of Economic Studies*, 83(2):810–854, 2016.

[31] Otto Kässi and Vili Lehdonvirta. Do digital skill certificates help new workers enter the market? evidence from an online labour platform. Working paper, CESifo, 2019.

[32] Otto Kässi and Vili Lehdonvirta. Online labour index: Measuring the online gig economy for policy and research. *Technological forecasting and social change*, 137:241–248, 2018.

[33] Jeroen Smits. GDL Area Database. GDL Working Paper 16-101, Global Data Lab, Institute for Management Research, Radboud University, Nijmegen, 2016. URL `https://globaldatalab.org/`.

[34] Daniel E. Russ, Kwan-Yuet Ho, Joanne S. Colt, Karla R. Armenti, Dalsu Baris, Wong-Ho Chow, Faith Davis, Alison Johnson, Mark P. Purdue, and Margaret R. Karagas. Computer-based coding of free-text job descriptions to efficiently identify occupations in epidemiological studies. *Occup Environ Med*, 73(6):417–424, 2016.

[35] David H. Autor, Frank Levy, and Richard J. Murnane. The skill content of recent technological change: An empirical exploration. *The Quarterly journal of economics*, 118(4):1279–1333, 2003.

[36] Alan S. Blinder. How many US jobs might be offshorable? *World Economics*, 10(2):41, 2009.

[37] Carl Benedikt Frey and Michael A Osborne. The future of employment: How susceptible are jobs to computerisation? *Technological forecasting and social change*, 114:254–280, 2017.

[38] Steven Tadelis, Xiaolan Zhou, et al. Buying reputation as a signal of quality: Evidence from an online marketplace. Technical report, National Bureau of Economic Research, 2016.

[39] Steven Tadelis. Reputation and feedback systems in online platform markets. *Annual Review of Economics*, 8:321–340, 2016.

[40] Lingfang Li, Steven Tadelis, and Xiaolan Zhou. Buying reputation as a signal of quality: Evidence from an online marketplace. *The RAND Journal of Economics*, 51(4):965–988, 2020.

[41] Maik Hesse, David Dann, Fabian Braesemann, and Timm Teubner. Understanding the platform economy: signals, trust, and social interaction. In *Proceedings of the 53rd Hawaii International Conference on System Sciences*, 2020.

[42] John B. Burbidge, Lonnie Magee, and A. Leslie Robb. Alternative Transformations to Handle Extreme Values of the Dependent Variable. *Journal of the American Statistical Association*, 83(401):123–127, 1988.

[43] James Andrew Hilden-Minton. *Multilevel diagnostics for mixed and hierarchical linear models*. University of California, Los Angeles, 1995.

[44] Andrew Bell, Malcolm Fairbrother, and Kelvyn Jones. Fixed and random effects models: making an informed choice. *Quality & Quantity*, 53(2):1051–1074, 2019.

[45] Vili Lehdonvirta, Helena Barnard, Mark Graham, and Isis Hjorth. Online labour markets-levelling the playing field for international service markets? Oxford, 2014.

[46] Thomas L. Friedman. *The world is flat: A brief history of the twenty-first century*. Macmillan, 2005.

[47] Phillip Brown, Hugh Lauder, and David Ashton. *The global auction: The broken promises of education, jobs, and incomes*. Oxford University Press, 2010.

[48] Gordon L Clark, Maryann P Feldman, Meric S Gertler, and Dariusz Wójcik. Introduction economic geography in the twenty-first century. In *The new Oxford handbook of economic geography*. 2018.

[49] Paul Collier. *The future of capitalism: Facing the new anxieties*. Penguin UK, 2018.

[50] Ed Michaels, Helen Handfield-Jones, and Beth Axelrod. *The war for talent*. Harvard Business Press, 2001.

[51] Elizabeth G Chambers, Mark Foulon, Helen Handfield-Jones, Steven M Hankin, and Edward G Michaels III. The war for talent. *The McKinsey Quarterly*, (3):44, 1998.

[52] Schon Beechler and Ian C Woodward. The global "war for talent". *Journal of international management*, 15(3):273–285, 2009.
