# Supplementary material for: The global polarisation of remote work
Source: PLoS One. 2022 Oct 20;17(10):e0274630. doi: 10.1371/journal.pone.0274630 (PMC9584402; doi:10.1371/journal.pone.0274630)
Supplement: S1 Text — (PDF) [file pone.0274630.s002.pdf]

Supporting Information

Supporting Information. This document contains additional information on the manuscript containing the following elements: *Overview and structure of the supporting information.* This part of the Supporting Information provides an overview of all additional materials and information provided in the appendix. *Conceptualising Remote Platform Work.* This element of the appendix discusses different definitions, interpretations and conceptualisations of remote platform work. We place the platform we investigate in the empirical part of the study in that classification. *Empirical approaches to measure platform work.* Here, we provide a tabular overview of the the main findings from the empirical literature related to our work, i. e. studies that measured the geography and role of skills in platform work. *Data collection and processing.* This part of the appendix provides all the details necessary to replicate the data collection and pre-processing of the data prior to analysis, including the collection and preparation of the online platform data, the regional statistical data, and occupation data; geocoding; and the derivation of occupation-level measures from the data. *Regression analysis of geographical polarisation.* Here, we outline the preparatory steps undertaken before conducting the regression analysis. In particular, we provide information about transformations and descriptive statistics about the output and explanatory features, and we discuss the choice of the final model specification used in the regression analysis. *Additional analyses.* This section presents robustness checks and further analyses. We investigate the robustness of the results with regard to the imputation of missing variables, analyse the model residuals, discuss the spatial granularity of the data, compare the wage distribution across countries and occupations, and we analyse the spatial polarisation over time.
